# Biological and thermochemical conversion of human solid waste to soil amendments

**DOI:** 10.1016/j.wasman.2019.04.010

**Published:** 2019-04-15

**Authors:** Leilah Krounbi, Akio Enders, Harold van Es, Dominic Woolf, Brian von Herzen, Johannes Lehmann

**Affiliations:** a Soil and Crop Sciences, School of Integrative Plant Science, College of Agriculture and Life Sciences, Cornell University, Ithaca, NY 14853, USA; b Atkinson Center for a Sustainable Future, Cornell University, Ithaca, NY 14853, USA; c Climate Foundation, Woods Hole, Massachusetts, MA 02543, USA

**Keywords:** Biochar, Compost, Feces, Sanitation, Sewage, Urine

## Abstract

Biological and thermochemical sanitization of source-separated human solid waste (HSW) are effective technologies for unsewered communities. While both methods are capable of fecal pathogen sterilization, the agronomically-beneficial properties of waste sanitized between methods remains unclear. Therefore, this study compared recovery and quality of soil amendments produced by compostation, torrefaction, and pyrolysis of HSW, established their financial value, and quantified tradeoffs between product value and conversion efficiency. Temperature and associated mass losses significantly affected the physical and chemical properties of thermochemically-treated HSW. Thermophilic composting, a biological sanitation method practiced in informal settlements in Nairobi, Kenya, produced an amendment that contained between 16 and 858-fold more plant-available nitrogen (N; 214.5 mg N/kg) than HSW pyrolyzed between 300 and 700 °C (0.2–15.2 mg N/kg). Conversely, HSW pyrolyzed at 600 °C had four-fold higher plant-available phosphorus (P; 3117 mg P/kg) and five-fold higher plant-available potassium (K; 7403 mg K/kg) than composted HSW (716 mg P/kg and 1462 mg K/kg). Wide variation between international fertilizer prices on the low end and regional East African prices on the high end resulted in broad-spaced quantiles for the value of agronomic components in HSW amendments. Phosphorus and K comprised a disproportionate amount of the value, 52–87%, compared to plant-available N, which contributed less than 2%. The total value of treated HSW, summed across all agronomic components per unit weight amendment, was greatest for thermochemically-treated HSW at 600 °C, averaging 220 USD/Mg, more than four-fold that of composted HSW, 53 USD/Mg. In contrast, torrefaction provided the highest monetary value per unit weight feedstock, 144 USD/Mg, as low heating temperatures engender minimal mass loss and higher nutrient densities per unit weight feedstock, compared to composted or pyrolyzed HSW. When benchmarked against total N, P, and K of eight commonly-applied organic amendments, including sewage-sludge (Milorganite), compost, and alfalfa meal, HSW pyrolyzed at 700 °C was of greatest value per unit weight of amendment, 365 USD/Mg, compared to 89 USD/Mg for composted HSW, and contained 2.9% total N (0.5 mg available N/kg), 3.1% total P (7640 mg available P/kg), 3.5% total K (17,671 mg available K/kg).

## Highlights

• Monetary value based on agronomic components was highest with HSW pyrolyzed at 600 °C.
• HSW pyrolyzed at 700 °C was of greatest monetary value compared to organic amendments.
• High mass recovery with torrefaction maximizes monetary value per unit weight feedstock.
• Compost had the lowest value under any scenario.

## 1 Introduction

Waste management is a growing concern globally. Nearly every aspect of our human lives produces waste, and rapid urban population growth is turning cities into nutrient sinks (Grimm et al., 2008). Furthermore, the lack of sewerage within high-density informal settlements leads to indiscriminate dumping of human solid waste (HSW) and urine, exacerbating nutrient loading into the environment (Drechsel et al., 2010, Gwenzi et al., 2015, Semiyaga et al., 2015). Less than half of the HSW excreted in lower- and middle-income cities such as Accra, Hanoi, and Bangkok is properly sanitized (Koné and Strauss, 2004). Effective, safe, and affordable waste management in cities should both mitigate human exposure to xenobiotics and pathogens while recovering valuable nutrients for agricultural production.

As the supply of mined phosphorus (P) dwindles (Cordell and White, 2014), and environmental and economic costs of synthetic nitrogen (N) increase (Canfield et al., 2010, Pikaar et al., 2017), urban latrine waste constitutes an untapped opportunity for nutrient recycling. Containing approximately 50% carbon (C), 4–5% N, 2–3% potassium (K), and 2–3% P (Rose et al., 2015, Onabanjo et al., 2016), ‘night soil’, or untreated human waste, has been land-applied for centuries to preserve soil fertility and bolster crop yields (Ferguson, 2014). Even today, untreated fecal wastes are directly land-applied for crop production, for example, in Ghana and Vietnam, albeit unadvisedly and at the expense of gastrointestinal health (Cofie et al., 2005, Jensen et al., 2008). Sociocultural and scientific perspectives on HSW in agriculture are now being revisited with the development of new technologies which facilitate safe and sanitary repurposing of HSW (Grant et al., 2012, Lopez-Rayo et al., 2016, Tobias et al., 2017, Simha et al., 2018).

Adequate HSW sanitization can be achieved through biological or thermochemical treatment. Thermophilic composting, a biological method in practice within informal settlements in Haiti (Berendes et al., 2015) and Sub Saharan Africa (Katukiza et al., 2012, Mawioo et al., 2016), relies on temperatures >60 °C to eliminate fecal pathogens (Vinnerås, 2007). Even in tropical climates, temperature variability within the compost pile poses a challenge to sterilization (Anand and Apul, 2014); composting operations in Port-Au-Prince, Haiti were lengthened by 4 weeks due to lower temperatures by 20 °C on pile corners relative to the center of the pile (Berendes et al., 2015). Furthermore land requirements for scaling up composting to serve entire cities are prohibitively large in informal settlements (Mawioo et al., 2016). More effective in assuring sanitization are thermochemical methods which treat HSW at 200–800 °C and require less than an hour to reach 500 °C at a ramp rate of 10 °C/min (Enders et al., 2012). Thermochemical technologies such as pyrolysis are being explored for decentralized HSW management (Katukiza et al., 2012, Gwenzi et al., 2015), yet direct comparisons of biological and thermochemical methods for optimum sterilization and resource recovery are lacking.

Amendments created from HSW may provide value to farmers in several ways, including provision of plant nutrients (fertilizer), increased nutrient use efficiency (thereby reducing future fertilizer costs), alleviating soil pH constraints to crop production, and providing alternative potential income streams such as carbon credits. Up to 58–70% of N in HSW is mineralizable to plant-available forms through composting (Hotta and Funamizu, 2007, Hotta et al., 2007), whereas high temperature thermochemical treatment ‘fixes’ N in cyclic aliphatic and heterocyclic aromatic forms which are not plant-available (Almendros et al., 2003, Wang et al., 2012). Conversely, with increasing highest heating temperature (HHT; the highest temperature reached during treatment), the concentration of ash nutrients including P and K rises. Alkalinity and liming potential are also enhanced as HHT increases (Enders et al., 2012). In tropical regions with acidic soils such as Kenya, P fertilizer and lime are cost-prohibitive and underapplied, increasing the value of higher-temperature, thermochemically-treated HSW (Opala et al., 2014). Nevertheless, a comprehensive assessment of the nutrient value of thermochemical products in comparison to composts is not available.

The value of HSW amendments extends beyond the concentration of nutrients directly supplied to include those retained in the soil due to adsorption on amendment surfaces. Cation exchange capacity (CEC) was found to be greater with lower-temperature thermochemical treatment of biomass, promoting greater nutrient retention in soils (Ippolito et al., 2012). Carbon stabilization is another valuable property with respect to prolonging the response to N and P fertilizers (Steiner et al., 2007), as well as carbon markets. Seventy to eighty percent of fecal C is respired as CO_2_ during thermophilic composting (Hotta et al., 2007), whereas high temperature thermochemical treatment stabilizes C in condensed aromatic compounds with mean residence times orders of magnitudes greater than the material from which they are produced (Lehmann et al., 2015).

Organic alternatives to mineral inputs are as useful as they are marketable. Farmers’ willingness to pay for organic amendments is contingent upon their performance relative to chemical fertilizers, as well as their quality relative to commercial organic amendments with which farmers have experience (Danso et al., 2006). Nutrient inventories of commercial organic amendments alongside market prices are useful for benchmarking their potential as alternatives to mineral fertilizers (Quilty and Cattle, 2011). Yet amendment prices may reflect processing and disposal costs rather than the concentration of plant-available nutrients, which is the main interest to end users. Our research explores the monetary value of HSW from the perspective of both farmers and waste-processors.

To generate a soil amendment from HSW with high agronomic and economic value, we sought to (1) compare recovery of soil amendments produced by compostation, torrefaction, and pyrolysis, (2) assess the concentration of plant-available nutrients, nutrient retention, liming potential, and persistent C in amendments, as well as toxicity factors including pathogens, heavy metals, organic contaminants, (3) establish their financial value, and (4) quantify the tradeoff between product value and conversion efficiency during processing.

We expected low temperatures and mildly oxidative conditions of the biological treatment to preserve the greatest amount of plant-available N, P, and K, generating the highest fertilizer-equivalent value per kg unsanitized HSW (HSW feedstock) to both farmers and the waste-processing operation. Additionally, higher mass recovery associated with lower temperatures was expected to generate larger volumes of marketable product, increasing the overall value of the processing. Among thermochemically-treated HSW amendments, higher rather than lower temperatures were expected to increase concentrations of non-volatile nutrients such as P and K, subsequently raising the fertilizer-equivalent value per unit weight of (sanitized) HSW to farmers. We also expected higher total N, P, and K concentrations leading to higher fertilizer-equivalent value in HSW amendments compared to commercially marketed organic amendments.

## 2 Materials and methods

### 2.1 Human solid waste collection and sanitation treatment

Soil amendments were produced from HSW collected from Sanergy Fresh Life latrines (Sanergy latrine manufacturing company, 2017) within the informal settlement of Mukuru in Nairobi, Kenya in March – June 2014. Sanergy latrine units utilize urine-diversion squat plates to separate urine from solid waste. HSW contained sawdust of exotic tree species: eucalyptus (*E. grandis, E. s*aligna*)*, pine (P. ponderosa, P. sylvestris, P. patula), cypress (*C. lusitanica*), and grevillea (*G. robusta*), added by latrine users as a cover material in a ∼1:1 vol ratio. We will continue to refer to the 1:1 mixture of HSW and sawdust used in this study as ‘HSW’. Daily HSW generation rates and data from chemical analyses refer to the mixture of HSW and sawdust.

We have also included in the supplementary information the concentration of agronomically-beneficial components in HSW not mixed with sawdust, and pyrolyzed at 300 °C, 400 °C, and 500 °C (Supplementary Table 1). For this, we obtained HSW from 10 Fresh Life latrines in which sawdust was not provided as a cover material. All drying, processing, and chemical analytical methods were identical to standard latrine HSW mixed with sawdust. This was not possible for composts as sawdust is required as bulking material. In addition, sawdust is needed for the latrines to operate. Therefore, comparisons in this study were made on the basis of HSW with sawdust to avoid bias.

Three kilograms (kg) of (wet) raw HSW (with sawdust) were randomly sampled from ten waste barrels the day after removal from latrines in Mukuru. Prior to thermochemical treatment, the initial gravimetric water content of HSW, 72.0 ± 6.8%, was lowered to below 30% through sun-drying, and thoroughly mixed. To prevent insect infestation during drying, waste was spread over plastic tarpaulins and covered with fitted mesh screens. HSW was then heated under anoxic conditions to 200 °C (torrefaction) and 300 °C, 400 °C, 500 °C, 600 °C, and 700 °C (pyrolysis), within a muffle furnace (Fisher Isotemp Model 126, Thermo Fisher Scientific, Waltham, MA) fitted with a drum and rotating paddle. To minimize air entry and oxidation, HSW was contained within a closed vessel in the furnace and continually swept with argon. All thermochemical treatments, torrefaction through pyrolysis, fully desiccated the HSW feedstock. The furnace temperature ramped up at 2.5 °C/min, characteristic of ‘slow pyrolysis’ conditions, to the highest heating temperature (HHT), at which it was held for 30 min. After the 30-minute dwell time, the furnace was shut off, and water piped through a coil around the heating vessel to accelerate cooling of the charred material.

HSW was sanitized biologically through thermophilic composting at a former waste-processing site within Mukuru. We used the Sanergy compost product, bagged for sale, for this research. Composting was carried out by Sanergy within wooden crates open at the bottom and top. During the thermophilic stage, once the compost pile reaches 60 °C, HSW was layered with additional carbonaceous materials, rice hulls and sugar cane residues. In the curing stage, box contents were piled into windrows and regularly turned. Both composting stages were carried out on bare earth over a period ranging between six to nine months. Prior to bagging, finished compost was filtered through a magnetic sieve (Circular Grid Magnet, Eclipse Magnets, Sheffield, England).

### 2.2 Measurements of the agronomic properties of HSW

Analysis of the agronomic properties of HSW amendments including nutrient content and soil conditioning potential were conducted on duplicate samples. Values were expressed per unit weight amendment in Table 3 and per unit weight feedstock after normalization by the mass yield of each temperature treatment (Supplementary Table 2). Plant-available N, ammonium (NH_4_^+^) and nitrate (NO_3_^–^), were extracted from amendments with 2M potassium chloride (KCl) at a ratio of 0.1 g/mL and analyzed colorimetrically on an autoflow analyzer (AA3 HR AutoAnalyzer, Seal Analytical, Mequon, WI). Plant-available macronutrients, P, K, calcium (Ca), magnesium (Mg), and sulfur (S), and micronutrients, boron (B), copper (Cu), manganese (Mn), zinc (Zn), were determined through extraction with Mehlich-III at a ratio of 0.1 g/mL (Mehlich, 1984). The supernatant was analyzed by inductively-coupled plasma optical emission spectroscopy (ICP-OES; Spectro Arcos, Ametek Materials Analysis, Kleve, Germany).

**Table 1:** Daily generation of HSW (including sawdust) measured in Sanergy Fresh Life latrines March–June 2014 and estimated urine production (Schouw et al., 2002, Rose et al., 2015).

Fresh HSW (g/person/day)	Water content (g/g)	Dry HSW (g/person/day)	Urine (mL/person/day)
161.3	0.7	48.4	1000

**Table 2:** Theoretical HSW and urine generation in Nairobi, based on waste generation measured in Sanergy Fresh Life latrines (Table 1).

	Nairobi	Nairobi informal settlements	Mukuru informal settlement	Sanergy latrine users 2015^a^
Population^b^	3,375,000	2,193,750	255,094	54,300
Dry HSW (Mg/community/day)	163.3	106.2	12.3	2.6
Urine (m^3^/community/day)	3375.0	2193.8	255.1	54.3

a Assuming 1086 Fresh Life latrines with 50 users per latrine in 2015.

b Population data from the Kenya census (KNBS, 2010) and the African Population and Health Research Center (APHRC, 2014).

**Table 3:** Concentration of agronomically-beneficial components in HSW amendments. Agronomic components include plant-available N (NH_4_^+^ + NO_3_^–^), P, K, Ca, Mg, S, micronutrients (B, Cu, Mn, Zn) reserve plant-available K^+^, Ca^2+^, and Mg^2+^ retained through CEC, CaCO_3_ equivalency, and BC_+100_. Data are the average of two measurements ± standard deviation.

Highest heating temperature (°C)								
Agronomic component	Unit	60 (compost)	200	300	400	500	600	700
N (NH_4_^+^+ NO_3_^–^)	mg/kg amendment	429 ± 2	780 ± 5	26.3 ± 2.0	11.6 ± 1.4	4.5 ± 4.8	2.1 ± 0.3	0.5 ± 0.0
P	g/kg amendment	1.44 ± 0.09	7.70 ± 0.52	6.65 ± 0.32	7.18 ± 0.45	8.15 ± 0.43	9.17 ± 0.58	7.64 ± 0.30
K	g/kg amendment	2.93 ± 0.15	1.42 ± 0.45	15.1 ± 0.59	17.3 ± 0.73	19.9 ± 0.60	21.8 ± 0.59	17.7 ± 0.53
Ca	g/kg amendment	5.12 ± 0.28	3.72 ± 0.21	2.86 ± 0.16	3.26 ± 0.35	3.51 ± 0.10	2.80 ± 0.17	4.16 ± 0.15
Mg	g/kg amendment	1.37 ± 0.08	4.23 ± 0.29	4.04 ± 0.18	4.32 ± 0.29	5.66 ± 0.38	6.43 ± 0.44	4.28 ± 0.16
S	mg/kg amendment	171 ± 1	520 ± 30	176 ± 12	220 ± 19	261 ± 20	298 ± 20	320 ± 6
Micronutrients (B + Cu + Mn + Zn)	mg/kg amendment	355 ± 18	297 ± 14	138 ± 5	171 ± 10	207 ± 10	237 ± 11	252 ± 7
CEC (K^+^ + Ca^2+^ + Mg^2+^)	g/kg amendment	9.56 ± 0.16	4.82 ± 0.31	9.43 ± 0.52	7.94 ± 0.27	6.20 ± 0.41	3.75 ± 0.33	4.19 ± 0.24
CaCO_3_	%w/w amendment	4.0 ± 1.3	0.5 ± 0.7	2.0 ± 0.2	4.9 ± 0.6	7.3 ± 0.2	5.6 ± 0.0	1.0 ± 0.6
BC_+100_	%w/w amendment	2.7 ± 3.4	11.2 ± 3.7	44.1 ± 2.1	60.5 ± 0.6	77.1 ± 3.4	85.2 ± 0.9	92.9 ± 1.0

The potential CEC was measured by saturating samples with NH_4_^+^ pH-adjusted to 7 and extracting with 2M KCl. Plant-available N in KCl-extracts was determined colorimetrically on an autoflow analyzer. The contribution of CEC to the retention of plant-available (Mehlich-III extractable) cations, K^+^, Ca^2+^, and Mg^2+^ present in HSW amendments was then calculated according to Eq. (1), based on the molar weight (Mw) and valence of each element *i*.

(1) $$\text{Cation}\;\text{retention}\;\text{(mg/kg)}=\frac{Mehlich\;III\;concentration\;of\;{cation}_{i}}{Mehlich\;III\;concentration\;\sum_{i}^{3}K^{+},{Ca}^{2+},{Mg}^{2+}}\times \frac{CEC}{{Valence}_{i}}\times {Mw}_{i}$$

The acid-neutralization potential of HSW amendments relative to calcium carbonate (CaCO_3_), also known as CaCO_3_ equivalency (% amendment/CaCO_3_), was measured by titration with sodium hydroxide (NaOH) after acidification with hydrochloric acid (HCl; Ahern et al., 2004). Persistent C, expressed as the proportion of C expected to remain in soil for over 100 years (BC_+100_) at 20 °C, was calculated as a function of the hydrogen (H) to organic C molar ratio (H/C_org_; Budai et al., 2013, Lehmann et al., 2015) based on incubation experiments conducted at 30 °C (Zimmerman, 2010) and 22 °C (Singh et al., 2012).

(2) $${BC}_{+100}\left(\%w/w\right)=-61.6\times H/C_{org}+105$$

Total C and H were measured by dry combustion (Flash 1112, CE Elantech; Ithaca, NY) and inorganic C (C_inorg_) was measured with a Bernard Calcimeter (Lamas et al., 2005) which measures the volume of carbon dioxide (CO_2_) emitted from a sample treated with 4 M HCl. Organic C (C_org_) is the difference between total and C_inorg_.

### 2.3 Toxicity analysis of HSW

Toxicity arising from fecal pathogens, heavy metals, polyaromatic hydrocarbons (PAH), polychlorinated biphenyls (PCB), and polychlorinated dibenzo-p-dioxins (PCDD) and dibenzofurans (PCDF) were measured in HSW amendments. HSW in various stages of sanitization, raw, sun-dried, autoclaved, composted, torrefied, and pyrolyzed, was aerobically cultured and analyzed for the following fecal pathogens with light microscopy: *Salmonella* species, *Shigella* species, *Aeromonas* species, *E. coli* serotype 157, *Yersinia enterocolitica*, and intestinal ova and parasites (Pathologists Lancet Kenya, Nairobi, Kenya).

Total heavy metal concentrations, cadmium (Cd), chromium (Cr), copper (Cu), nickel (Ni), lead (Pb), and zinc (Zn) were evaluated using ICP-OES after digestion with perchloric and nitric acids (Hseu, 2004) on an automated digestion block (Vulcan, Questron Technologies Corp., Mississauga, ON).

Pace Analytical labs (Schenectady, NY) conducted the PAH analysis following a soxhlet extraction (Environmental Protection Agency (EPA) Methods 8270D, 3540C). Analyses for PCBs and PCDD/Fs were conducted according to EPA Methods 1668A and 8290A, respectively. The toxicity of PCDD/Fs is expressed in toxicity equivalents (TEQ), calculated as the sum over the concentration of each PCDD/F congener weighted by its toxic equivalency factor (TEF; Eq. (3)). We relied on the World Bank’s 2005 estimates of TEF values per PCDD/F (Van den Berg, 2006).

(3) $$TEQ=\sum C_{i}X{TEF}_{i}$$

### 2.4 HSW amendment value according to two approaches

Two approaches were used for assessing the monetary value of HSW-derived soil amendments: (1) a ‘bottom-up’ approach in which the sum of incremental contributions of ten agronomic components was equated to the overall value of HSW amendments (2) a ‘top-down’ approach, benchmarking the value of HSW as a bulk amendment against commercial soil amendments based on their market prices and total N, P, and K concentrations.

For the first approach, the value of HSW amendments in US dollars (USD) per megagram (Mg) dry, unsanitized feedstock or per kg sanitized amendment, was calculated as the concentration of each agronomic component (*i*) multiplied by its market price, summed over all components (Eq. (4)).

(4) $$\text{HSW}\;\text{amendment}\;\text{(USD/Mg)}=\sum_{i}^{n}\left({agronomic\;component}_{i}\;x\;{component\;price}_{i}\right)$$

Market values for fertilizer nutrients were taken from the Africa Fertilizer Information Portal (africafertilizer.org, 2018) for dates between February 2016 and June 2017. Prices were aggregated from East African commodity prices listed as ‘National’, as well as international commodity prices, ‘International’ (Supplementary Table 3). Prices for fertilizers containing sulfur (S), magnesium (Mg), and micronutrients B, Cu, Mn, and Zn, were taken from AliBaba (https://www.alibaba.com/, 2017). Agricultural lime (CaCO_3_) prices were taken from two cement companies in Kenya, Arm Cement and Rhino Cement, as well as CaCO_3_ commodities listed on AliBaba. Both East African and international CaCO_3_ prices were used for estimating the Ca value in amendments. The value of cation retention potential was calculated as the proportion of the plant-available fraction of three exchangeable cations, K^+^, Ca^2+^ and Mg^2+^ retained on amendment surfaces via CEC using their market prices (Eq. (1)). The value of persistent organic C (BC_+100_) was determined using the 2015 discount rates for CO_2_ across 23 countries (World Bank Group, 2015).

The monetary value of each agronomic component was aggregated into five quantiles (*n* = 0.1, 0.25, 0.5, 0.75, 0.9) based on at least ten price values spanning International (low end, quantiles 0.1–0.5) and East African (high end, quantiles 0.5–0.9) prices. Each quantile *n* * 100 represents prices at and below the *n*% of the entire price range for each agronomic component. The sensitivity of the total value of HSW amendments, was expressed as the difference between maximum and minimum quantile values, summed over ten agronomic components in HSW amendments.

For the second, ‘top-down’ approach, the value of HSW per kg amendment was benchmarked against eight commercial soil amendments: animal manure, compost, vermicompost, Milorganite, soybean meal, alfalfa meal, cottonseed meal, and bone meal, based on the most common metric for assessing amendment quality: total N, P, and K contents (Quilty and Cattle, 2011; Supplementary Table 4, Supplementary Table 5). We inversely solved for the nutrient price of total N, P, and K in amendments using the Excel ‘solver’ tool (Excel Solver, Fylstra et al., 1988) by minimizing an objective function (OF) of the difference between the amendment market price and the sum over the product of each nutrient concentration and nutrient price (*i*_1_ = N, *i*_2_ = P, and *i*_3_ = K; Eq. (5)). Two conditions were specified for OF calculations: (1) fixed price ratios for P/N; and (2) fixed price ratios for P/K, according to single-nutrient fertilizer prices used in the ‘bottom-up’ approach (Supplementary Table 3) and taken from the Africa Fertilizer Information Portal (africafertilizer.org, 2018). Objective functions were calculated for each of the five market price quantiles (*k* = 0.1, 0.25. 0.5, 0.75, 0.9); the average nutrient price (*k* = 0.5) was used to assess HSW amendment price.

(5) $${OF}_{k}\;\text{= min[}{market\;price\;of\;amendment}_{k}\;\text{-}\sum_{i}^{n=3}\left({nutrient\;concentration}_{i}\;{X\;nutrient\;price}_{i}\right]$$

The average bulk HSW amendment value was then equated to the sum of the average (*k* = 0.5) value of total N, P, and K concentrations, *i*_1_ = N, *i*_2_ = P, and *i*_3_ = K, in amendments (Eq. (6)).

(6) $$\text{Ave.}\;\text{bulk}\;\text{HSW}\;\text{amendment}\;\text{(USD/Mg)}=\sum_{i}^{n=3}\left({nutrient\;concentration}_{i}\;{X\;nutrient\;price}_{i,k=0.5}\right]$$

### 2.5 Statistics

Exploratory data analysis, graphics, and linear regressions were carried out with the *ggplot2* package (Wickham, 2009) in R statistical computing language (R Core Team, 2017). Boxplots show the inter-quantile range (IQN), spanning across 0.25–0.75 quantiles, with the center line representing the median value, the lower hinge the 0.25 quantile, and the upper hinge the 0.75 quantile. Statistical outliers, shown as points, are defined as greater than 1.5*IQN, above and below the lower hinges. In this research, statistical outliers were only identified above the 1.5*IQN.

Linear regression coefficients, goodness of fit (R^2^), and p values were calculated and plotted using the *lm()* function and *summary.lm()* method. Linear regression along the line of best fit for quantile values of each agronomic component in HSW amendments was carried out using the *quantreg* package (Koenker, 2018), with its default modified version of the Barrodale and Roberts algorithm for *l1*-regression (Koenker and d'Orey, 1987). Additional R packages employed for this research are listed in Supplementary Table 6.

## 3 Results

### 3.1 Mass recovery and chemical properties of human solid waste

The amount of dry HSW generated in Sanergy latrines per person per day in 2014 averaged 48.4 g (Table 1; Sanergy, personal communication, 2017). This value falls within the daily dry fecal weight range for 57 low income households reported by Rose et al. (2015), 18–62 g/person/day. At this rate, 12.3 Mg HSW per day is expected for the broader Mukuru neighborhood in which Sanergy is located, and 106.2 Mg per day for the more than two million people residing within Nairobi’s informal settlements (Table 2). Biological and thermochemical processing methods effectively sanitized HSW, as no fecal pathogens were detected in any of the processed HSW samples (Supplementary Table 7).

**Fig. 1:**
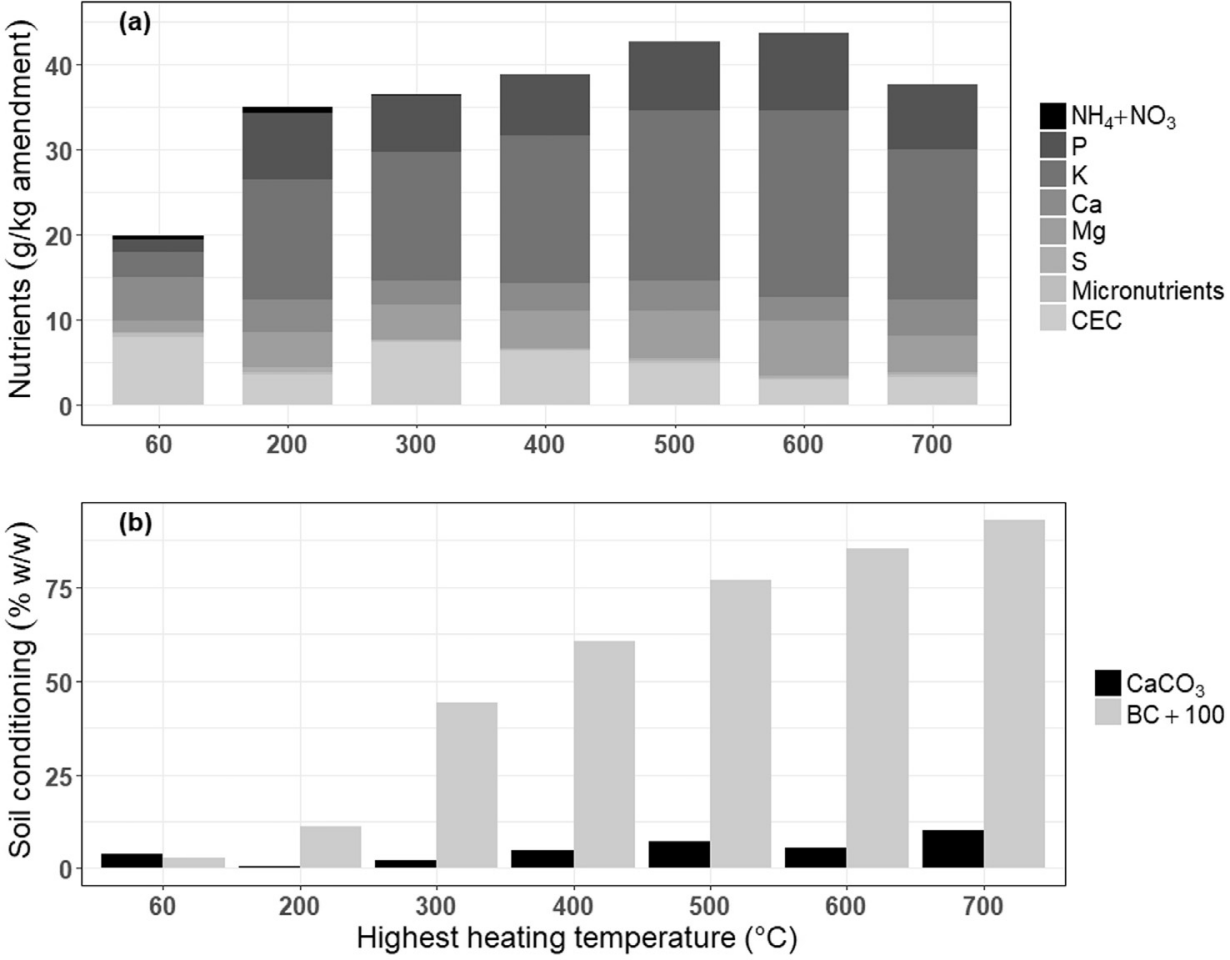
(a) Plant-available nutrients in biologically (60 °C compost) and thermochemically-treated HSW amendments: N (NH_4_^+^ NO_3_^–^), P, K, Ca, Mg, S, micronutrients (B, Cu, Mn, Zn), and the contribution of the CEC in retaining plant-available K^+^, Ca^2+^, and Mg^2+^ (b) CaCO_3_ equivalency and BC_+100_. Values are averages of duplicate measurements (Table 3).

Mass recovery expectedly decreased with increasing HHT, with up to 60% (w/w) mass reduction at 700 °C and only 10% at 200 °C (Supplementary Fig. 1). The physical and chemical properties of HSW amendments were also affected by HHT (Fig. 1, Table 3, Supplementary Table 2). Compared to 600 °C pyrolyzed HSW, torrefied (200 °C) HSW contained 371-fold greater plant-available N per kg amendment (1002-fold greater per kg feedstock), but only 0.84-fold available P per kg amendment (2.2-fold per kg feedstock), and 0.65-fold available K per kg amendment (1.7-fold per kg feedstock). HSW pyrolyzed at 300 °C had the largest CEC per kg amendment, corresponding to 2.5-fold more base cations (K^+^ + Ca^2+^ + Mg^2+^) available long term than when pyrolyzed at 600 °C (3.9-fold per kg feedstock), and 2-fold more base cations than torrefied HSW (1.2-fold per kg feedstock). The ratio of hydrogen to organic C (H/C_org_) of HSW amendments from which BC_+100_ is calculated, revealed that 85.2% of C_org_ in 600 °C pyrolyzed HSW versus only 11.2% of C_org_ in torrefied HSW is expected to remain in soil for 100 years. On a per-kg feedstock basis accounting for 40 and 90% mass recovery from biological or thermochemical conversion (Supplementary Fig. 1), HSW pyrolyzed at 600 °C provides 2.9-fold more BC_+100_ than torrefied HSW, respectively (Fig. 1, Table 3, Supplementary Table 2).

To compare amendment quantity across the thermochemical temperature spectrum, one day’s worth of HSW collected in Nairobi’s informal settlements and treated via torrefaction, generates approximately 96 Mg dry HSW/day containing 75.5 kg available N, 735.5 kg available P, 1357.9 kg available K, and 11.7 Mg of persistent C. Conversely, pyrolysis at 600 °C produces only 36 Mg dry HSW/day containing 0.1 kg available N, 330.9 kg available P, 785.9 kg available K, with 30.7 Mg of persistent C. Amendment quality, however, follows a different trend. If one ton of each HSW amendment is land-applied, torrefied HSW can supply approximately 0.8 kg available N, 7.7 kg available P, 14.2 kg available K, and 5 kg of CaCO_3_-equivalency. These values increase with additions of 600 °C pyrolyzed HSW, which is expected to contribute 9.2 kg available P, 21.8 kg available K, and 55.6 kg of lime-equivalency (Supplementary Fig. 1, Table 2, Table 3).

Heavy metal concentrations in all HSW amendments did not exceed threshold limits set by the U.S. EPA or by the Austrian Compost Ordinance for use in conventional agriculture or land reclamation (Table 4). Toxic heavy metals Cd, Cr, Ni, and Pb did not increase with increasing HHT. Composted HSW and HSW pyrolyzed at 500 °C contained between 3.8 and 14-fold greater amounts of Cd, Cr, and Ni compared to other amendments (Table 4). Lead levels were greatest in composted HSW, 50.0 mg/kg, above the threshold for use in organic agriculture (Austrian Compost Ordinance; Amlinger et al., 2004, Hogg et al., 2002). The concentration of Ni in 500 °C HSW was 5.0 and 2.6-fold greater than values reported for dry excreta in Thailand, 4.5 mg/kg, or Sweden, 8.7 mg/kg (Schouw et al., 2002, Vinnerås et al., 2006). Chromium was also 4.2-fold greater in 500 °C HSW compared to values for Sweden (5.4 mg/kg) while lead values in 500 °C HSW were higher than those reported in Thailand, 6.5 vs. 1.0 mg/kg, but lower than values for Sweden, 36.5 mg/kg.

**Table 4:** Total acid-digestible heavy metals in HSW amendments alongside acceptable threshold concentrations for biosolids (U.S. EPA) and compost (Austria) intended for land-application (mg/kg dry mass).

	Highest heating temperature (°C)							EPA	Austrian compost ordinance^b^		
	60 (compost)	200	300	400	500	600	700	Biosolids CCL^a^	Class A organic ag.	Class A agriculture	Class B land reclamation
Metal	(mg/kg amendment)						(mg/kg dry mass)				
Cd	0.43 ± 0.18	0.15 ± 0.00	0.23 ± 0.01	0.27 ± 0.01	0.43 ± 0.05	0.22 ± 0.05	0.03 ± 0.00	85	0.7	1	3
Cr	23.6 ± 1.3	2.8 ± 0.4	12.1 ± 2.2	5.0 ± 0.1	22.5 ± 0.5	5.9 ± 0.7	4.0 ± 0.2		70	70	250
Cu	42 ± 2	31 ± 2	76 ± 2	72 ± 5	107 ± 6	395 ± 265	153 ± 2	4300	70.00	150	450
Ni	18.3 ± 1.0	4.7 ± 0.2	11.9 ± 0.8	9.1 ± 0.0	22.3 ± 1.0	8.6 ± 0.7	10.4 ± 0.1	420	25	60	100
Pb	50.0 ± 1.8	6.1 ± 4.7	4.4 ± 0.1	4.7 ± 0.5	6.5 ± 0.7	3.5 ± 1.3	4.7 ± 0.2	840	45	120	200
Zn	280 ± 5	237 ± 6	374 ± 27	470 ± 48	484 ± 55	591 ± 30	760 ± 12	7500	200	500	1500

a Ceiling Concentration Limits (CCL) EPA Section 503.13 (1995).

b Amlinger et al., 2004, Hogg et al., 2002.

Conversely, Cu and Zn, which are also essential plant micronutrients, did increase with HHT, and were 3.5-fold and 2-fold greater in 500 °C HSW compared to torrefied HSW. Both Cu and Zn in 500 °C HSW were above threshold for use in organic agriculture (Amlinger et al., 2004, Hogg et al., 2002), reaching 106.7 mg Cu/kg and 483.5 mg Zn/kg. Copper and Zn, micronutrients in trace amounts, were also elevated in 500 °C HSW compared to fresh, dry HSW from Thailand or Sweden (1.3–4.8-fold Cu and 1.3–3.6-fold Zn). All values except Cd were higher in composted HSW (0.43 mg/kg) than values reported by Vinnerås et al. (2006) for Sweden (0.51 mg/kg) or Schouw et al. (2002) for Thailand (0.51 mg/kg).

Between 16.5 and 30-fold greater amounts of PAHs were measured in thermochemically-treated HSW at 300 °C and 500 °C compared to composted HSW and HSW pyrolyzed at 700 °C (Table 5, Supplementary Table 8). Regardless, the highest PAH concentration was measured in HSW pyrolyzed at 500 °C, 1633 μg/kg, and was 73% lower than the European PAH toxicity threshold, 6000 μg/kg (European Commission, 2001). All amendments had similarly low concentrations of PCBs and PCDD/Fs, 0.91–2.59 μg/kg and 0–0.97 ng/kg, respectively, measuring two orders of magnitude below European and American toxicity thresholds (Table 5, Supplementary Table 9, Supplementary Table 10; EPA, 2000, European Commission, 2001, Fürhacker and Lence, 1997).

**Table 5:** Total PAH, PCB, and PCDD/F concentrations in HSW amendments alongside toxicity thresholds.

	Highest heating temperature (°C)				Toxicity thresholds		
Contaminant	60 (compost)	300	500	700	European Commission^a^	Lower Austria^a^, ^b^	EPA^c^
PAH^d^ (µg/kg amendment)	56	942	1633	54	6000		
PCB (µg/kg amendment)	1.96^e^	0.91^e^	1.22^e^	2.59^e^	800^f^	200^ g^	
PCDD/F TEQ^h^ (ng/kg amendment)	0.97	0	0	0	100	100	300

a European Commission (2001).

b Fürhacker and Lence (1997).

c EPA (2000).

d Sum of acenaphthene, benzo(a)pyrene, benzo(b,k)fluoranthene, benzo(g,h,i)perylene, fluoranthene, fluorene, indeno(1,2,3-cd)pyrene, phenanthrene, pyrene (Supplementary Table 8).

e Sum of PCB congeners 1–209 (Supplementary Table 9).

f Sum of PCB congeners 28, 52, 101, 118, 138, 153, 180.

g Sum of PCB congeners 28, 52, 101, 138, 153, 180.

h Sum of TEQ for all PCDD/F congeners (Supplementary Table 10).

### 3.2 Monetary value of human solid waste amendments

#### 3.2.1 Value as sum of agronomic components, ‘bottom-up’ approach

**Fig. 2:**
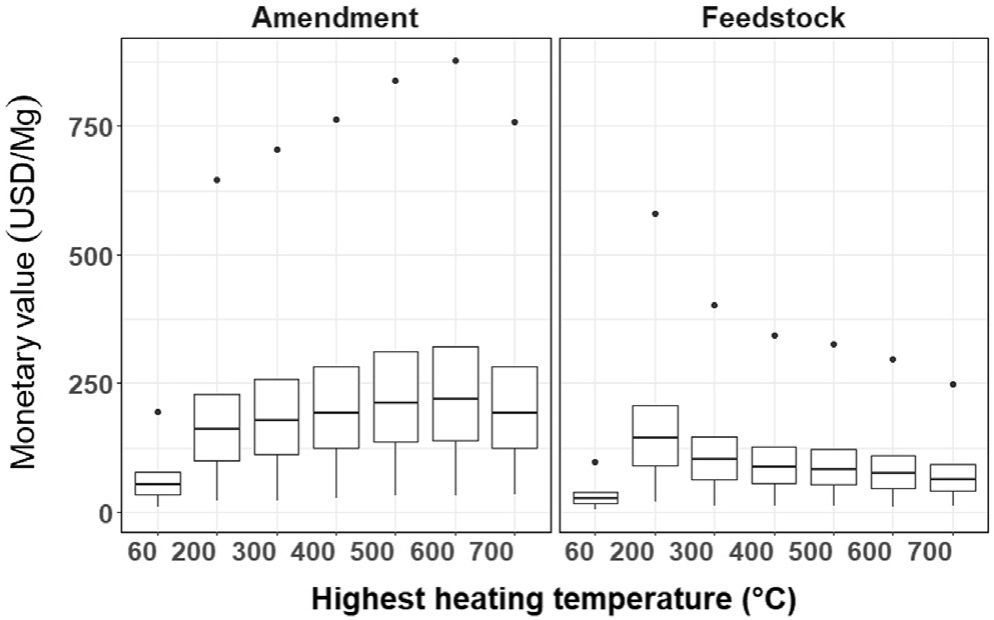
Five quantiles of the monetary value in USD of biologically (60 °C compost) and thermochemically-treated HSW amendments per megagram (Mg) of dry weight of sanitized HSW (Amendment) and unsanitized HSW (Feedstock), based on ten agronomic components. Value of agronomic components is based on nutrient content of HSW (Table 3, Supplementary Table 2) and market prices for each component (Supplementary Table 3).

When expressed per unit weight of untreated feedstock, torrefied HSW was valued at 144.2 USD/Mg feedstock, 1.9-fold more than 600 °C pyrolyzed HSW. Per unit weight of amendment, this trend was reversed: 600 °C pyrolyzed HSW was worth 220.0 USD/Mg amendment, 1.4-fold more than torrefied HSW. Composted HSW had the lowest monetary value per unit weight of feedstock and per unit weight of amendment: 26.4 USD/Mg feedstock, 140.2–446.2% lower than thermochemically-treated HSW and 52.7 USD/Mg amendment, 204.0–317.5% lower than thermochemically-treated HSW (Fig. 2, Supplementary Table 11).

**Fig. 3:**
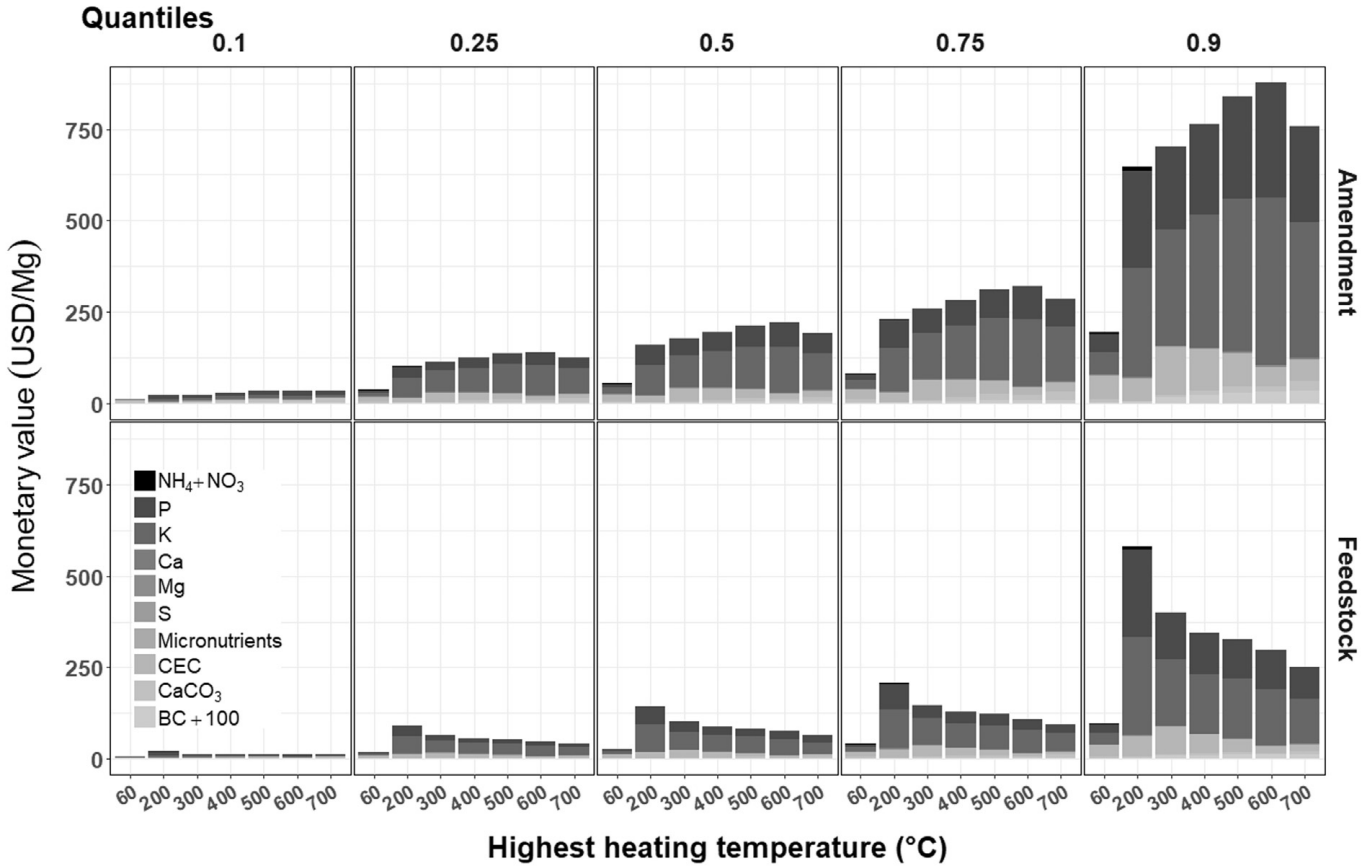
Quantile monetary values in USD per unit weight of amendment (top row) and per unit weight of feedstock (bottom row), for agronomically-beneficial components in biologically (60 °C compost) and thermochemically-treated HSW. Agronomic components include plant-available N (NH_4_^+^+ NO_3_^–^), P, K, Ca, Mg, S, micronutrients (B, Cu, Mn, Zn), and the contribution of the CEC toward retention of plant-available K^+^, Ca^2+^, and Mg^2+^, CaCO_3_ equivalency, and BC_+100_. Values are based on nutrient content in HSW (Table 3, Supplementary Table 2) and market prices of agronomic components (Supplementary Table 3).

**Fig. 4:**
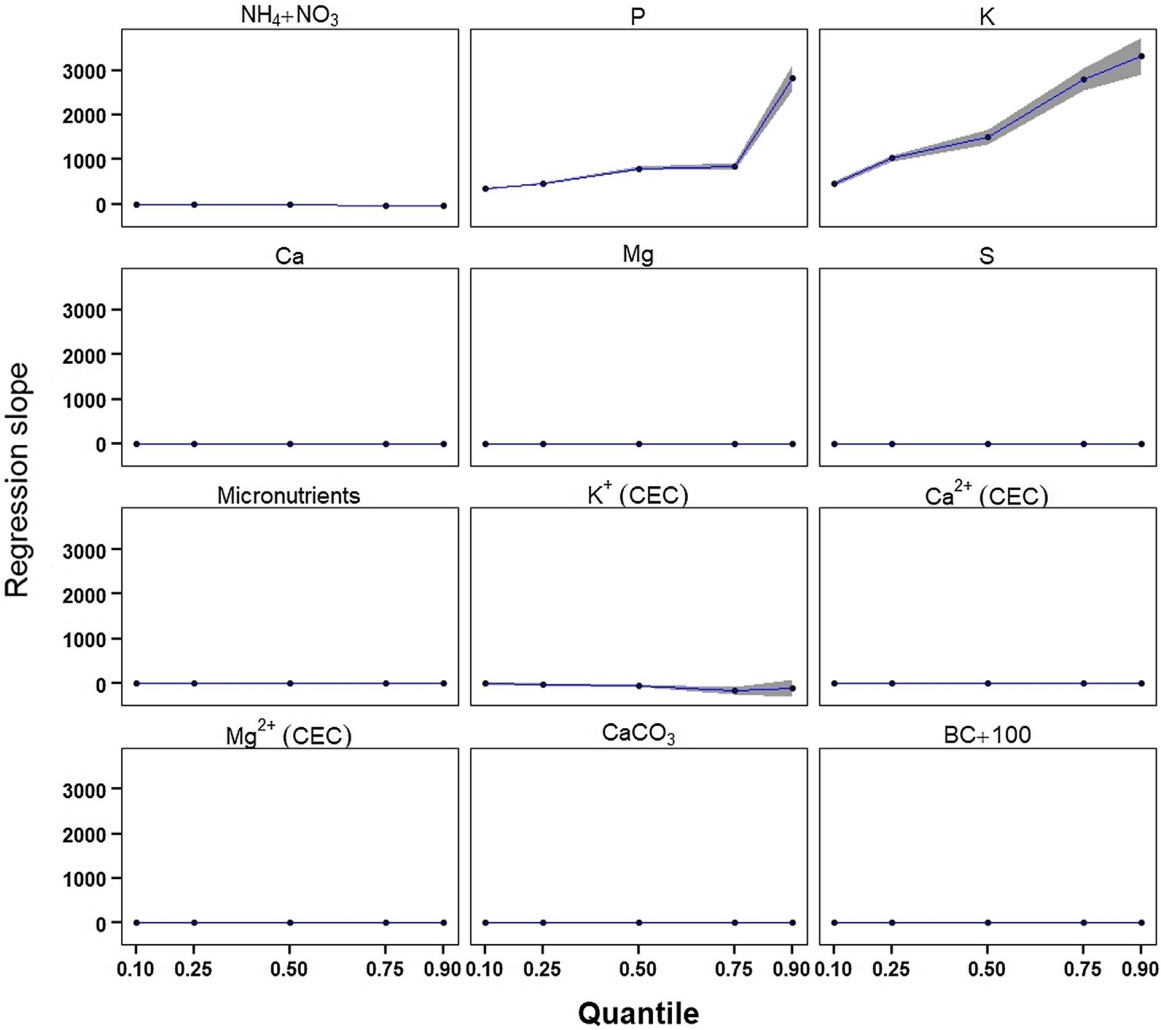
The change in the slope (β) of the regression of HSW amendment value versus HHT, plotted as a function of price quantiles for agronomic components including plant-available N (NH_4_^+^+ NO_3_^–^), P, K, Ca, Mg, S, micronutrients (B, Cu, Mn, Zn), the retention of K^+^, Ca^2+^, and Mg^2+^ estimated by the CEC, CaCO_3_ equivalency, and BC_+100_. Quantile regression coefficients of HSW amendment value vs. HHT are listed in Supplementary Table 12.

Large margins between East African (quantiles 0.5–0.9) and international prices (quantiles 0.1–0.5) fertilizer prices created wide-spaced price quantiles for the monetary value of HSW amendments (Fig. 3). The difference between the maximum and minimum quantile values (in USD per unit weight of amendment) spanned between 624.5 and 844.7 USD/Mg for thermochemically-treated HSW (Table 6) while the maximum quantile value (p = 0.9) was a statistical outlier for all amendments (Fig. 2). At the lowest quantile (p = 0.1), P and K contributed 54–81% to the value of thermochemically-treated HSW, while it rose to 77–88% at the largest quantile (p = 0.9). Quantiles regression of the values of each agronomic component in HSW over HHT revealed significant temperature effects of P and K at higher quantile values; the slopes of the regression line for P and K were 8.3- and 7.3-fold steeper at quantile 0.9 compared to quantile 0.1 (Fig. 4, Supplementary Table 12).

**Table 6:** Price sensitivity of HSW amendments by agronomic component expressed as the difference between the 0.9 and 0.1 quantile prices.

	Highest heating temperature (°C)						
Agronomic component	60 (compost)	200	300	400	500	600	700
	Quantile 0.9 – quantile 0.1^a^ (USD/Mg amendment)						
NH_4_^+^ + NO_3_^–^	5.7	10.4	0.36	0.15	0.06	0.03	0.01
P	47.5	254.6	220.1	237.4	269.6	303.3	252.7
K	60.1	291.9	310.4	355.7	409.1	447.2	362.9
Ca	1.4	1.01	0.78	0.89	0.95	0.76	1.10
Mg	0.6	1.9	1.8	2.0	2.6	2.9	2.0
S	0.01	0.03	0.01	0.01	0.02	0.02	0.02
Micronutrients (B + Cu + Mn + Zn)	0.21	0.25	0.11	0.12	0.17	0.28	0.55
CEC (K^+^ + Ca^2+^ + Mg^2+^)	61.3	59.8	128.8	110.4	86.6	52.3	56.7
CaCO_3_	5.60	0.71	2.87	6.92	10.30	7.90	14.20
BC_+100_	0.9	3.9	15.5	21.3	27.1	30.0	32.7

a Quantile market prices for macro- and micronutrients, CaCO_3_, and C are listed in Supplementary Table 3.

#### 3.2.2 Value as bulk amendment compared to commercial products, ‘top-down’ approach

**Fig. 5:**
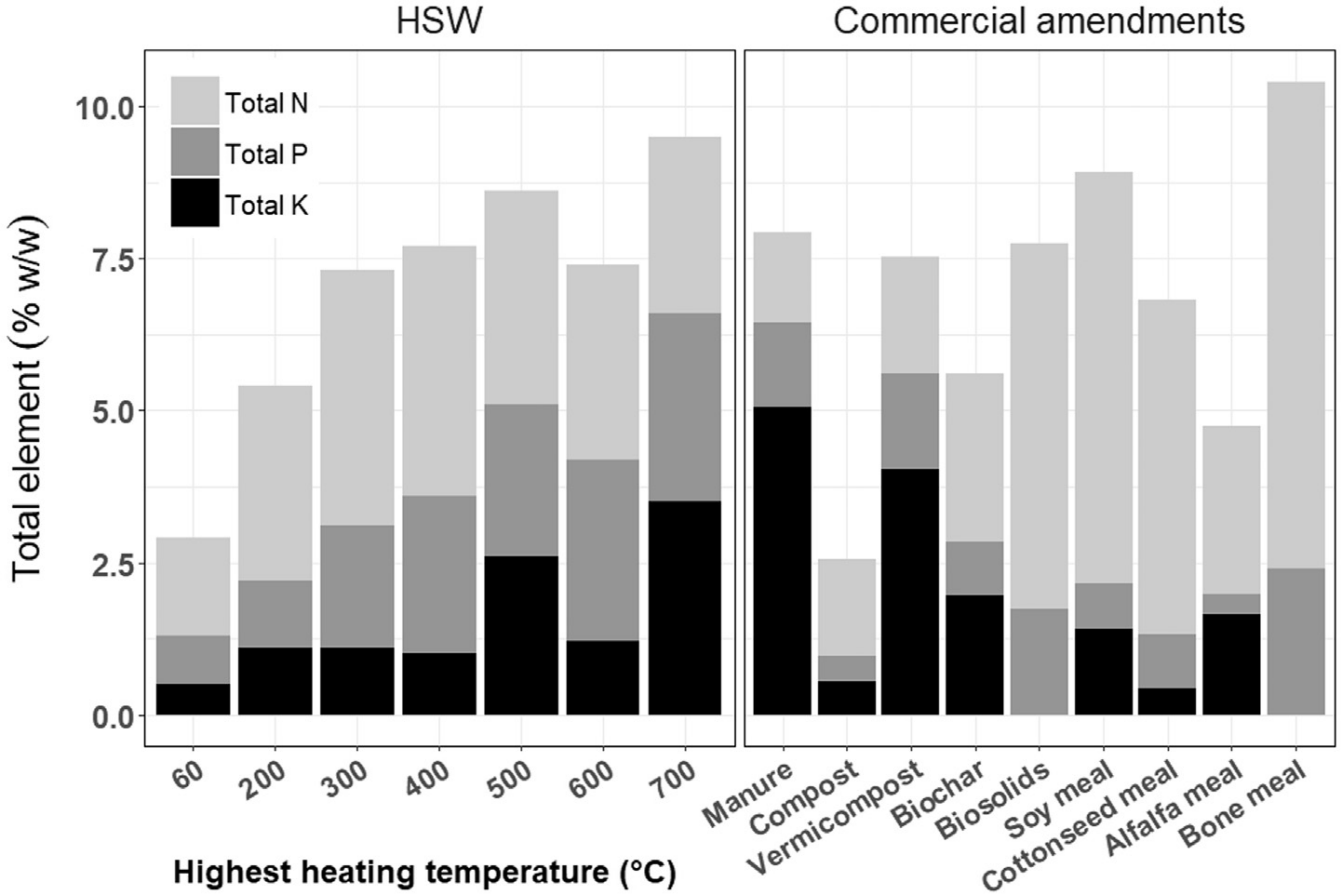
Total concentration of N, P, and K in biologically (60 °C compost) and thermochemically-treated HSW amendments compared to nine commercial soil amendments (Supplementary Table 4, Supplementary Table 5).

**Fig. 6:**
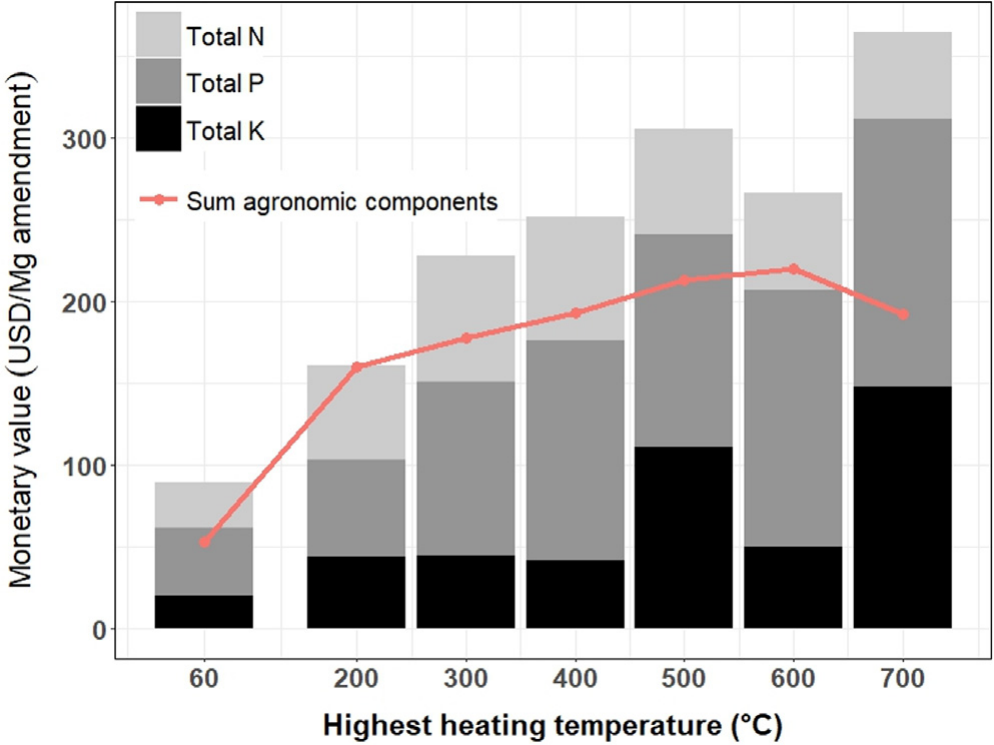
The monetary value of biologically (60 °C compost) and thermochemically-sanitized HSW amendments calculated by two methods. Bars represent show the ‘top down’ approach, HSW value benchmarked that of eight commercial amendments. The line plot shows the median value of HSW amendments summed over agronomic components, per unit weight of amendment, according to the ‘bottom-up’ approach (Fig. 2, Supplementary Table 11).

The sum of total N, total P, and total K was comparable between thermochemically-treated HSW and commercial soil amendments excluding commercial compost (Fig. 5, Supplementary Table 4, Supplementary Table 5). Total N, P, and K ranges of commercial compost were similarly low as HSW compost. The value of HSW amendments calculated using a ‘top-down’ approach, benchmarked against commercial amendments, was greater than the ‘bottom-up’ approach for all amendments except torrefied HSW (Fig. 6, Supplementary Table 11, Supplementary Table 13). The ‘top down’ approach put 700 °C pyrolyzed HSW at 324.5 USD Mg/amendment, 2-fold greater than torrefied HSW, 143.0 USD Mg/amendment. The sensitivity of HSW amendment value to processing HHT varied with commercial amendment type. Commercial biochar, derived primarily from plant biomass, had the highest market price and showed the steepest change in value with increasing HHT, followed by alfalfa meal (Supplementary Fig. 2). It was not included in the top-down assessment of HSW value due to its rarity as a commercial product and its unusually high price.

## 4 Discussion

With increasing use of organic amendments to enhance soil quality, assessments on their value to farmers has centered on feedstock type (Quilty and Cattle, 2011, Chen et al., 2018). Biomass-based soil amendments such as plant residues and animal manure can be applied with little pre-treatment, making feedstock type the only variable in the discussion of amendment quality and value. In contrast, HSW must be sanitized before land-application, the multiple options for which considerably alter amendments from the original feedstock. The properties of composted, torrefied, and pyrolyzed HSW discussed in this research were as disparate as those between cattle manure, food waste, and alfalfa meal (Supplementary Table 5).

### 4.1 Resource recovery

Each sanitization method showed different mass conversion efficiencies, affecting the final nutrient composition of amendments as well as the amount of marketable product. The greater mass recovery at lower versus higher pyrolysis temperatures can be explained by lower losses of volatilizable elements such as C, N, H, O, and S during thermochemical conversion (Enders et al., 2012, Ippolito et al., 2015, Zhang et al., 2015). Nevertheless, the concentration of volatilizable nutrients was lower in composted than torrefied HSW (Krounbi et al., 2018) despite the lower composting temperature, 60 °C vs. 200 °C.

Unlike pure heat-based sterilization achieved by heating waste >100 °C, pathogen elimination via thermophilic composting relies on heat generated through intensive microbial respiration, resulting in CO_2_-C and water losses (Tiquia et al., 2002). In their study of C and N emissions during feces composting, 80% of feedstock C was respired as CO_2_ (Hotta and Funamizu, 2007), over three times greater gaseous emissions than reported by Yacob et al. (2018) during pyrolysis of human fecal waste (17.2–29.6%) at varying ramp rates. Moreover, while CO_2_ was emitted in highest proportion, 29–58%, during pyrolysis between 300 and 450 °C, H_2_ comprised over 50% of gas emissions at 600–700 °C. Methane and nitrous oxide emissions can also be lowered if biomass such as HSW is thermochemically-processed rather than composted; Gaunt and Cowie (2012) estimated the potential for averting 1.47 and 0.20 kg of CO_2_-equivalents per kg biomass in the form of methane and nitrous oxide through pyrolysis instead of composting.

In addition to C, N mineralized during the composting process may also be lost through volatilization as NH_3_ (Hotta and Funamizu, 2007), as well as through leaching; prolonged exposure of HSW compost to the ambient atmosphere likely resulted in leaching losses of NO_3_^–^ and base cations Ca^2+^, Mg^2+^, and K^+^ (Eghball et al., 1997, Bernal et al., 2017, Onwosi et al., 2017). In addition to greater overall resource recovery as C and mineral nutrients, thermochemical treatment likely resulted in less greenhouse gas emissions compared to composting.

### 4.2 Product quality

All treatment methods evaluated in our research successfully converted HSW into a safe and beneficial soil amendment based on EU and U.S. EPA environmental standards. Biological sanitation and low-temperature thermochemical treatments created amendments similar to commercial products such as manure, composts, or alfalfa meal. Thermophilic composting and torrefaction preserved C and N in mineralizable forms, as processing conditions are not favorable for condensing of aliphatic C into C=C bonds that is observed during pyrolysis (Baldock and Smernik, 2002, McBeath et al., 2015). This is also shown by low BC_+100_ calculated for both materials in comparison to the biochars. The increase in base cations and P with increasing HHT is likely due to losses of other elements, O and H during the conversion of aliphatic C to aromatic C.

Composted and torrefied HSW were vastly different from each other in spite of similarly lower HHT than those known to creating fused aromatic ring structures. To initiate microbial thermophilic degradation of pathogens in HSW, the C/N ratio was raised (Bernal et al., 2017, Onwosi et al., 2017) with additions of rice hulls and bagasse (Sanergy, personal communication, 2017). These materials were not added to feedstock before pyrolysis. Other factors not pertaining to the composting process may have affected the quality of HSW compost. Strong signatures of soil admixtures are apparent in the high contents of heavy metals which could have resulted from composting on a bare earthen floor, as turning and handling the compost are part of normal operations. Moreover, high Pb levels in HSW compost may have resulted from dust deposition from the industrial area of Mukuru in Nairobi, where Sanergy formerly processed all latrine waste (operations have since moved to the rural locale of Kinanie, South of Nairobi). Even if leaded gasoline and paint are not in use in Nairobi, re-suspension in the atmosphere of older Pb-contaminated dust can be a persistent source of contamination in cities (Del Rio-Salas et al., 2012).

Heavy metals were more concentrated in composted HSW and slightly more concentrated in thermochemically-treated HSW compared to those reported for fresh, dry HSW from Thailand and Sweden (Schouw et al., 2002, Vinnerås et al., 2006); both studies considered HSW to be overly enriched in toxic elements Cd or Zn, and Pb. The Thai study attributed higher than expected Cd levels to the staple food rice, reportedly enriched in Cd naturally (Ikeda et al., 2000). The Swedish study explained that galvanized Zn pipes and Pb pipes may have raised levels of both elements above the expected. Thus, the presence of heavy metals in HSW may be indicative of baseline environmental contamination present also in other purposeful and incidental soil amendments such as animal manure, irrigation water, even dust. A study from Wales and the UK found atmospheric deposition as the main contributor (25–85% of total inputs) to heavy metal accumulation in soils. They also found more than double the contribution of Zn, Cu, and Cd to soils from application of livestock manure compared to biosolids (Nicholson et al., 2006).

Furthermore, mineral phosphate (P_2_O_5_) fertilizers contain heavy metals, and were noted to contribute up to 74% to the total Cd load in arable land across Europe (EUROSTAT, 1995, de Meeus et al., 2002). A study which evaluated 196 P_2_O_5_ fertilizers sold across Europe for heavy metals (Nziguheba and Smolders, 2008) found similar concentrations compared to HSW: 7.4 (Cd), 2.9 (Pb), 166 (Zn) mg/kg. The caveat lies in the much higher HSW amendment application rates to supply the same amount of P per hectare (ha); approximately 1.5 Mg/ha of 700 °C HSW is needed to supply 40 kg P (total P; Supplementary Table 4), compared to only 92 kg/ha P_2_O_5_. Based on values in Table 4 and those listed in Nziguheba and Smolders (2008) for P_2_O_5_, the annual Cd, Pb, and Zn contribution from 700 °C HSW would be 0.05 (Cd), 7.05 (Pb), 1140 (Zn) g/ha while inorganic P_2_O_5_ fertilizers would contribute 0.68 (Cd), 0.27 (Pb), 15.2 (Zn) g/ha. However, if organic inputs such as the chicken (layer) manure analyzed in Nicholson et al. (2006) were land-applied at the same amount of 1.5 Mg/ha, toxic heavy metal loading would be greater than those from 700 °C HSW: 2.16 (Cd), 13.44 (Pb) mg/kg. Heavy metal loading is therefore lowest with commercial fertilizers (except for Cd) but is nevertheless lower in HSW amendments than commonly-applied animal manures.

In a farming system relying on organic inputs, charred amendments may have an advantage over uncharred amendments in their lower leachability of heavy metals in soils. Even as pyrolysis preserves the total, acid-digestible heavy metals in the biochar, resulting in larger concentrations with higher treatment temperatures, as shown for Zn and Cu in this research, the bioavailable fraction is typically reduced after pyrolysis. Lowered bioavailability with increasing pyrolysis temperature may even compensate for increased total contents. Jin et al. (2016) found increasing total contents of Cu, Zn, Cr, and Pb in sewage sludge pyrolyzed at 600 °C versus 400 °C. Yet the 600 °C sludge biochar posed the lowest ecological risk for all four metals due their reduced bioavailability compensating for overall concentrations, compared to the lower-temperature biochars. Devi and Soraha (2014) showed that over 40% of the total Cd, Pb, and Zn in paper-mill effluent sludge pyrolyzed at 700 °C was converted into non-bioavailable forms (neither water, acid, or base-extractable and not exchangeable). Biochar has also been reported to lower ambient heavy metal bioavailability already present in soils, as shown in the study by Park et al. (2011), in which application of pyrolyzed chicken manure (550 °C) to soil from a shooting range lowered bioavailable Cd by 94.7% and Pb by 99.9%. Thus, we expect low overall bioavailability of heavy metals in HSW biochars and the soils that they are applied to.

The low concentrations of potentially toxic substances (PAH, PCB, dioxin, heavy metals) in both composts and thermochemical products indicated that the studied materials do not pose a contamination hazard when applied to land. It is important to note that the low PAHs, dioxins, and PCBs produced during thermochemical conversion apply to the specific processing conditions utilized here, which included anoxic conditions, slow kiln ramp rate (<20 °C/min), and temperatures not above 700 °C (Hale et al., 2012, Wang et al., 2017).

Sanergy HSW compost and thermochemically-processed HSW used in our research was fully sterilized. Nevertheless, other studies have described difficulties in sterilizing HSW through thermophilic composting (Niwagaba et al., 2009, Lemunier et al., 2005, Piceno et al., 2017). Lemunier et al. (2005) observed *Salmonella serovar* Enteritidis colonies in mature, thermophilically-composted HSW after 12 weeks. Niwagaba et al. (2009) found that even in tropical Uganda, styrofoam insulation was required to achieve temperatures > 50 °C in HSW compositing bins. Therefore, thermochemical conversion methods are safer approaches to sterilization without drawbacks in nutrient recovery.

### 4.3 Commercial value

The strong sensitivity of amendment monetary value to P and K, both per unit weight of feedstock and per unit weight of amendment, highlights the attractiveness of HSW amendments enriched in these nutrients as fertilizer substitutes. High P prices in Sub-Saharan Africa are one cause of persistent underapplication, estimated at less than 30% of the total fertilizer use (Syers et al., 2011, Nziguheba et al., 2016). Nevertheless, the contribution of P applications to total P in soils in East Africa has been increasing rapidly, with projected increases in P fertilizer contribution to total P reaching 75% for 2050 (Mogollón et al., 2018). High-temperature pyrolyzed HSW can be used as a source of recovered plant-available P to meet the growing demand in regions such as in Eastern Africa.

Market accessibility to fertilizers is not, however, only a function of cost or proximity. Lime, for instance, is underapplied among Western Kenyan maize farmers suffering under soil acidity (Kiplagat et al., 2014, Opala et al., 2018), despite the commodity being mined by Homa Lime Co. LTD in the Nyanza province of Western Kenya (Homa lime Co. LTD, 2018, Yager, 2011). A similar argument can be made for the benefits of applying organic matter to build soil organic C. Touted as improving the yield response to mineral fertilizer (Marenya and Barrett, 2009, Güereña et al., 2016, Van Zwieten, 2018), the addition of organic matter is less cost-restrictive to farmers as it is labor-intensive, because the main expense in labor; organic C as residual waste biomass is relatively cheap to apply. Okalebo et al. (2006) found a 73% increase in maize yield after incorporation of lablab bean, compared to 116% increase with mineral fertilizer. The relative cost of each intervention, the lablab bean relay and mineral fertilizer, increased overall production costs by 6% and 69%, respectively. The advantage of an amendment such as 700 °C pyrolyzed HSW over multiple intervention strategies involving the purchasing of inputs in tandem with organic amendment procurement is that this one amendment is a concentrated source of multiple agronomically-beneficial properties, including available P, liming potential, and BC_+100_. And while CaCO_3_ equivalency and BC_+100_ were not significant contributors to HSW amendment values, they improve P availability (Nziguheba et al., 2000, Kisinyo et al., 2014, Krounbi et al., 2018), which has significant equivalent monetary value in HSW amendments. If multiple sanitation methods were utilized for treating HSW, one could create a combination fertilizer comprised of HSW pyrolyzed at 700 °C and torrefied HSW, able to supply farmers with P, lime, organic C, and mineral N, allowing farmers to tackle multiple soil quality problems with one amendment.

### 4.4 Tradeoff between product value and conversion efficiency

Different tradeoffs and advantages are apparent between low and high temperatures of thermochemical treatments. Greater net mass and nutrient recovery with lower treatment temperatures favored the value of torrefaction over pyrolysis from the perspective of the waste processers. This will also be a point of consideration in terms of global resource recovery to maximize nutrient return to soil. However, handling and transportation costs of the final product may significantly contribute to costs of operations (Roberts et al., 2010).

An assessment of two decentralized systems, one for treating waste from individual septic systems in wastewater treatment plants in Dakar, Senegal (Dodane et al., 2012), and the other for container-based sanitation relying on thermophilic composting in Cap Haiten, Haiti (Tilmans et al., 2015) revealed similar low costs per person, based on the daily (dry) HSW excretion rate per person reported by Sanergy of 48.4 g/person/day (Table 1). The system relying on trucking and treatment in waste water treatment plants was estimated to cost around USD 429/Mg dry HSW/yr or USD 0.02/person/day, while the container-based system relying on thermophilic composting in the same manner as Sanergy was estimated to cost USD 658/Mg dry HSW/yr which equates to USD 0.03/person/day.

Cost differences between thermochemical methods such as torrefaction or pyrolysis and composting arise from the purchase and maintenance of a continuous pyrolysis reactor. Woolf et al. (2017) estimated the cost of an industrial-scale reactor with a capacity of 250 kg biomass/hour at USD 580,000. The reactor described by Woolf et al. (2017) can process biomass with a moisture content up to 30% most efficiently between 450 and 600 °C, ideal conditions for HSW. Among thermochemical systems, pyrolysis at 500–600 °C has better energy efficiency than torrefaction (200 °C), gasification (>700 °C), or combustion in the combined energy density of recovered syngas, oil, and char (Lehmann, 2012, Samolada and Zabaniotou, 2014, Hanif et al., 2016). Utilizing the reactor proposed by Woolf et al. (2017) to sanitize HSW for 10 h a day, 7 days a week, for three years, at a capacity factor of 0.7, equates to USD 302.67/Mg dry HSW/yr, a value comparable to processing costs reported for both Senegal and Haiti (Dodane et al,. 2012, Tilmans et al., 2015).

Furthermore, a product with lower value per mass may not be economical, as in the case of poultry manure, in which C degradation was promoted to enhance the concentrations of valuable plant nutrients, N, P, and K (Penn et al., 2011). Another consideration is that products may be evaluated by end-users, in this case farmers, based on their value as soil amendments with respect to P or K. Those amendments with greater P concentrations per unit weight of amendment (700 °C pyrolyzed HSW) may have significant financial impacts, as Kenyan farmers pay 55–87% more for high P fertilizers such as triple super phosphate (TSP) than US farmers (Supplementary Table 3). Other soil quality constraints such as low soil organic C and high acidity, prevalent across soils in the tropics, are effectively ameliorated with pyrolyzed HSW compared to the torrefied or composted HSW.

## 5 Conclusion

Large potential for nutrient recovery lies in HSW conversion schemes. The thermochemical waste processing methods presented in this work solve both health and sanitation problems while providing a more concentrated source of plant nutrients in comparison to the tested composting. The success of waste recycling lies in the marketability of the final product that varied several fold between the biological and thermochemical conversion methods tested here. Waste-processors in urban areas stand to profit more from torrefaction due to higher mass recovery and high monetary value per kg feedstock. From the point of view of nutrient resource conservation, torrefaction makes more nutrients (especially plant-available N) but less persistent C available per kg feedstock than pyrolysis. In contrast, farmers located on acidic, C-deficient, and P-fixing soils may benefit more from higher P concentrations, the persistent C, and the lime-equivalency of 700 °C pyrolyzed HSW.

Our work relied on chemical extractions as proxies for plant-availability, but further work should determine actual crop yields as affected by HSW amendments. Further work should also determine both the immediate and long-term availability of heavy metals in HSW amendments to soil biota and plants (compared to alternate organic amendments and mineral P fertilizers), and greenhouse gas emissions associated with the different conversion strategies. Furthermore, our analysis of monetary value did not account for the entire value chain, including management logistics and costs at Sanergy and/or transportation of HSW amendments to rural farmers. We also did not directly ask farmers which amendment type they preferred. Further research is necessary to conclude which treatment method is indeed the most economical for the waste processors and which amendment is most attractive to farmers.

We have shown that sanitization of HSW through both composting and thermochemical processing mitigates exposure to pathogenic organisms while concentrating plant-essential nutrients. In areas lacking waste management such as Nairobi’s informal settlements, this would provide an important social health benefit while also generating a valuable fertilizer product. We conclude, therefore, that HSW sanitization can provide a valuable win-win outcome in which urban sanitation and agronomic efficiency both benefit from a potentially important way to close nutrient cycles and turn a problematic waste into a valuable fertilizer.
