# Supplementary material for: Biological and thermochemical conversion of human solid waste to soil amendments
Source: Waste Manag. 2019 Apr 15;89:366–78. doi: 10.1016/j.wasman.2019.04.010 (PMC6538828; doi:10.1016/j.wasman.2019.04.010)
Supplement: Supplementary data 1 [file mmc1.docx]

Supplementary online material

**Biological and thermochemical conversion of human solid waste to soil amendments**

Leilah Krounbi^1^, Akio Enders^1^, Harold van Es^1,2^, Dominic Woolf^1^, Brian van Herzen^3^, Johannes Lehmann^1,2*^

*^1^Soil and Crop Sciences, School of Integrative Plant Science, College of Agriculture and Life Sciences, Cornell University, Ithaca, NY 14853, USA*

*^2^Atkinson Center for a Sustainable Future, Cornell University, Ithaca, NY 14853, USA*

*^3^Climate Foundation, Woods Hole, Massachusetts, MA 02543, USA*

*corresponding author, Email: [CL273@cornell.edu](mailto:CL273@cornell.edu)


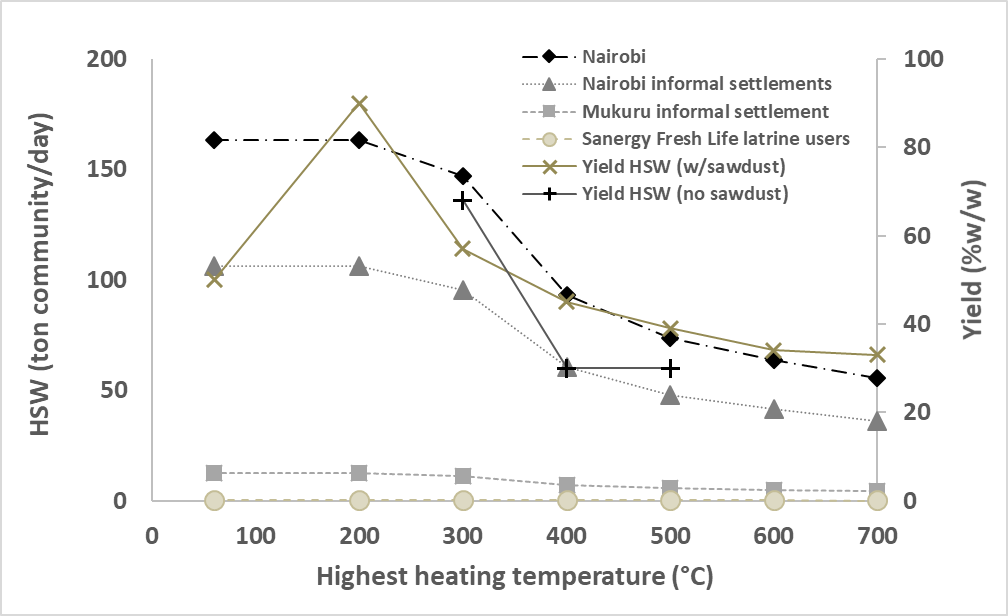


Supplementary Fig. 1. Theoretical mass recovery of human solid waste (HSW) mixed with sawdust (w/sawdust) from Nairobi and neighborhoods within Nairobi, Kenya following biological (60 °C compost) and thermochemical treatment at 200 – 700 °C, based on the measured yield recovery of torrefied and pyrolyzed Sanergy HSW (Yield; brown **🞨**). Also included is the yield of HSW not mixed with sawdust (no sawdust) pyrolyzed at 300 °C, 400 °C, and 500 °C (Yield; black 🞤).

Supplementary Table 1

Concentrations of agronomically-beneficial components in HSW not mixed with sawdust, and pyrolyzed at 300 °C, 400 °C, and 500 °C. Agronomic components include plant-available N (NH_4_^+^+ NO_3_^-^), P, K, Ca, Mg, S, micronutrients (B, Cu, Mn, Zn) reserve plant-available K^+^, Ca^2+^, and Mg^2+^ retained through CEC, CaCO_3_ equivalency, and BC_+100_. All values except BC+_100_ are the average of two measurements ± standard deviation.

| Highest heating temperature (°C) | | | | |
| --- | --- | --- | --- | --- |
| Agronomic component | Unit | 300 °C | 400 °C | 500 °C |
| N (NH_4_^+^+ NO_3_^-^) | mg/kg feedstock | 62.5 ± 1.1 | 3.8 ± 0.6 | 2.8 ± 1.9 |
| P | mg/kg feedstock | 3820 ± 60 | 3578 ± 89 | 3772 ± 306 |
| K | mg/kg feedstock | 16,923 ± 61 | 12,553 ± 139 | 11,888 ± 791 |
| Ca | mg/kg feedstock | 1274 ± 37 | 920 ± 25 | 1013 ± 49 |
| Mg | mg/kg feedstock | 2208 ± 187 | 2171 ± 51 | 2313 ± 37 |
| S | mg/kg feedstock | 439 ± 5 | 237 ± 7 | 238 ± 25 |
| Micronutrients (B+Cu+Mn+Zn) | mg/kg feedstock | 68.8 ± 3.1 | 56.0 ± 1.3 | 57.5 ± 2.6 |
| CEC (K^+^+Ca^2+^+Mg^2+^) | mg/kg feedstock | 4673 ± 65 | 2040 ± 135 | 1801 ± 4 |
| CaCO_3_ | %w/w feedstock | 1.7 ± 0.8 | 3.3 ± 0.3 | 3.0 ± 0.0 |
| BC_+100_ | %w/w feedstock | 17.5 (1 rep) | 22.8 (1 rep) | 23.3 (1 rep) |
| N (NH_4_^+^+ NO_3_^-^) | mg/kg amendment | 92.0 ± 1.6 | 12.7 ± 2.1 | 9.2 ± 6.2 |
| P | mg/kg amendment | 5617 ± 89 | 11,926 ± 298 | 12,574 ± 1017 |
| K | mg/kg amendment | 24,886 ± 90 | 41,845 ± 462 | 39626 ± 2637 |
| Ca | mg/kg amendment | 1874 ± 54 | 3065 ± 82 | 3377 ± 164 |
| Mg | mg/kg amendment | 3248 ± 55 | 7238 ± 169 | 7710 ± 622 |
| S | mg/kg amendment | 646 ± 7 | 790 ± 22 | 793 ± 85 |
| Micronutrients (B+Cu+Mn+Zn) | mg/kg amendment | 101 ± 5 | 187 ± 4 | 192 ± 9 |
| CEC (K^+^+Ca^2+^+Mg^2+^) | mg/kg amendment | 6939 ± 96 | 13,770 ± 448 | 12,864 ± 12 |
| CaCO_3_ | %w/w amendment | 2.5 ± 1.1 | 11.0 ± 0.9 | 10.2 ± 0.0 |
| BC_+100_ | %w/w amendment | 25.8 (1 rep) | 76.0 (1 rep) | 77.7 (1 rep) |

Supplementary Table 2

Concentrations of agronomically-beneficial components in HSW amendments per unit weight of feedstock. Agronomic components include plant-available N (NH_4_^+^+ NO_3_^-^), P, K, Ca, Mg, S, micronutrients (B, Cu, Mn, Zn) reserve plant-available K^+^, Ca^2+^, and Mg^2+^ retained through CEC, CaCO_3_ equivalency, and BC_+100_. Data are the average of two measurements ± standard deviation.

| Highest heating temperature (°C) | | | | | | | | |
| --- | --- | --- | --- | --- | --- | --- | --- | --- |
| Agronomic component | Unit | 60 (compost) | 200 | 300 | 400 | 500 | 600 | 700 |
| N (NH_4_^+^+ NO_3_^-^) | mg/kg feedstock | 214.5  ± 0.7 | 701.5  ± 4.1 | 15.2  ± 1.1 | 5.2  ± 0.6 | 1.8  ± 1.9 | 0.7  ± 0.1 | 0.2  ± 0.0 |
| P | mg/kg feedstock | 718  ± 47 | 6928  ± 472 | 3792  ± 184 | 3229  ± 204 | 3179  ± 169 | 3118  ± 197 | 2521  ± 100 |
| K | mg/kg feedstock | 1463  ± 74 | 12792  ± 405 | 8614  ± 338 | 7793  ± 331 | 7769  ± 236 | 7403  ± 202 | 5832  ± 176 |
| Ca | mg/kg feedstock | 2560  ± 138 | 3350  ± 190 | 1632  ± 89 | 1468  ± 156 | 1367  ± 41 | 952  ± 57 | 1372  ± 48 |
| Mg | mg/kg feedstock | 685  ± 41 | 3807  ± 260 | 2305  ± 101 | 1946  ± 133 | 2209  ± 150 | 2185  ± 150 | 1413  ± 54 |
| S | mg/kg feedstock | 85  ± 6 | 468  ± 27 | 100  ± 7 | 99  ± 9 | 102  ± 8 | 101  ± 7 | 106  ± 2 |
| Micronutrients (B+Cu+Mn+Zn) | mg/kg feedstock | 177.5  ± 8.8 | 267.2  ± 12.4 | 78.4  ± 3.0 | 77.1  ± 4.3 | 80.7  ± 4.0 | 80.6  ± 3.7 | 83.2  ± 2.4 |
| CEC (K^+^+Ca^2+^+Mg^2+^) | mg/kg feedstock | 4778  ± 81 | 4338  ± 278 | 5376  ± 296 | 3573  ± 120 | 2419  ± 159 | 1273  ± 113 | 1382  ± 78 |
| CaCO_3_ | %w/w feedstock | 2.0  ± 0.6 | 0.5  ± 0.6 | 1.2  ± 0.1 | 2.2  ± 0.3 | 2.8  ± 0.1 | 1.9  ± 0.0 | 3.3  ± 0.2 |
| BC_+100_ | %w/w feedstock | 1.3  ± 1.7 | 10.1  ± 3.4 | 25.1  ± 1.2 | 27.2  ± 0.3 | 30.1  ± 1.3 | 29.0  ± 0.3 | 30.7  ± 0.3 |


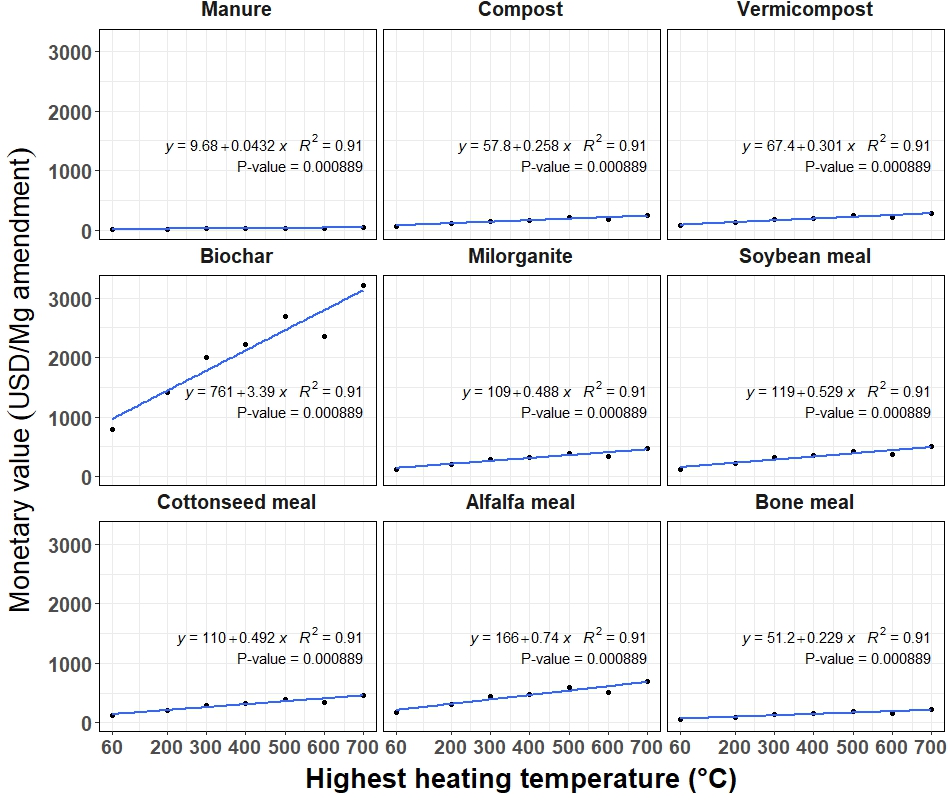


Supplementary Fig. 2. Linear regression between the monetary value of HSW amendments, calculated using the ‘top-down’ approach, versus the sanitization temperature. Monetary value is based on the comparative concentration of total N, P, and K in HSW (Supplementary Table 4) versus nine commercial organic amendments and their market prices (Supplementary Table 5).

Supplementary Table 3

International and East African market prices for fertilizer nutrients including N (NH_4_^+^+ NO_3_^-^), P, K, Ca, Mg, S, micronutrients (B, Cu, Mn, and Zn), as well as CaCO_3_, and carbon dioxide (CO_2_) discount rates. Prices in bold were used for calculating the final value HSW presented in Fig. 3.

| Source | Agronomic component | Quantile prices (USD/Mg) | | | | |
| --- | --- | --- | --- | --- | --- | --- |
|  |  | 0.1 | 0.25 | 0.5 | 0.75 | 0.9 |
| AfricaFertilizer.org; eight East African countries; February 2016 - June 2017 | NH_4_^+^+ NO_3_^-^-N | 1051 | 1877 | 2799 | 4018 | 13,668 |
|  | P | 2973 | 4870 | 7807 | 11,602 | 34,464 |
|  | K | 1426 | 4665 | 6690 | 9097 | 20,985 |
|  | Ca^a^ | 93.2 | 93.2 | 93.2 | 102.5 | 111.8 |
| AfricaFertilizer.org; International prices; February 2016 - June 2017 | NH_4_^+^+ NO_3_^-^-N | 383 | 464 | 512 | 1807 | 2013 |
|  | P | 1385 | 1528 | 1666 | 3652 | 4612 |
|  | K | 448 | 455 | 1167 | 2184 | 2424 |
|  | **S** | **396** | **407** | **438** | **450** | **463** |
|  | Ca | 140 | 157 | 217 | 336 | 366 |
| AfricaFertilizer.org; eight East Africa and International prices combined; February 2016 - June 2017 | NH_4_^+^+ NO_3_^-^-N | **384** | **1580** | **2534** | **3890** | **13,668** |
|  | **P** | **1385** | **3843** | **7238** | **9893** | **34,464** |
|  | **K** | **448** | **3862** | **5736** | **8423** | **20985** |
|  | **Ca** | **93.2** | **143.2** | **170.9** | **332.6** | **365.5** |
| Kenya cement companies and Alibaba, June 2017 | **CaCO_3_** | **50** | **105** | **119** | **235** | **261** |
|  | **Mg** | **207** | **359** | **406** | **460** | **663** |
| Alibaba, June 2017 | **B** | **743** | **3235** | **3801** | **4139** | **4730** |
|  | **Zn** | **1194** | **1266** | **1578** | **1860** | **2059** |
|  | **Mn** | **1199** | **1269** | **1368** | **1625** | **1692** |
|  | **Cu** | **5108** | **6631** | **7564** | **8291** | **10,216** |
| State and trends of Carbon (C) pricing 2015; World Bank Group | **CO_2_** | **1.0** | **6.5** | **16.0** | **32.0** | **130.0** |

^a^ CaCO_3_ was used for lime prices and Ca prices.

Supplementary Table 4

Acid-digestible total N, P, and K in sanitized HSW amendments. Data are the average of two measurements ± standard deviation.

| Highest heating temperature (°C) | Total N | Total P | Total K |
| --- | --- | --- | --- |
|  | (% w/w) | | |
| 60 | 1.6 ± 0.1 | 0.8 ± 0.2 | 0.5 ± 0.1 |
| 200 | 3.2 ± 0.6 | 1.1 ± 0.1 | 1.1 ± 0.2 |
| 300 | 4.2 ± 0.3 | 2.0 ± 0.0 | 1.1 ± 0.1 |
| 400 | 4.1 ± 0.1 | 2.6 ± 0.1 | 1.0 ± 0.1 |
| 500 | 3.5 ± 0.2 | 2.5 ± 0.1 | 2.6 ± 0.1 |
| 600 | 3.2 ± 0.0 | 3.0 ± 0.0 | 1.2 ± 0.1 |
| 700 | 2.9 ± 0.3 | 3.1 ± 0.0 | 3.5 ± 0.0 |

Supplementary Table 5

Literature values for the concentration of total N, P, and K in various organic amendments and their market prices.

| Description | N | P | K | Source | Material | Country | Cost | Source |
| --- | --- | --- | --- | --- | --- | --- | --- | --- |
|  |  | (%w/w) |  |  |  |  | (USD/Mg) |  |
| **Manure** | | | | |  |  |  |  |
| Zero grazed cattle manure | 1.7 | 1.85 | 2.05 | Njenga et al., 2010 | Farmyard manure | Kenya | 10 | Kirigia et al., 2013 |
| Average of composted and biodynamic FYM | 2.2 | 4.60 | 2.16 | Zaller and Koepke, 2004 | Farmyard manure | Kenya | 30 | Otinga et al., 2013 |
| Dairy manure and bedding | 1.5 | 0.04 | 0.76 | Carpenter-Boggs et al., 2000 | Cattle manure | USA | 35 | MSU, 2008 |
| Cattle manure | 1.0 | 0.22 | 0.44 | Penhallegon, 2003 | Farmyard manure | Kenya | 50 | Wango et al., 2015, 2016 |
| Dairy manure | 1.0 | 0.17 | 0.44 | Penhallegon, 2003 | Farmyard manure | Kenya | 100 | Nekesa et al., 2007 |
| **Compost** | | | | |  |  |  |  |
| MSW compost | 2.0 | 0.58 | 0.39 | Hargreaves et al., 2009a,b | Compost | Kenya | 3 | Wango et al., 2015, 2016 |
| Garden compost | 1.0 | 0.16 | 0.5 | Chan et al., 2007a | Compost | USA | 28 | Home Depot amendments |
| Urban waste compost | 1.2 | 0.45 | 0.6 | Njenga et al., 2010 | Compost | USA | 70 | Ideal compost co. |
| Greenwaste | 2.3 | 0.33 | 0.65 | Penhallegon, 2003 | Compost | USA | 50 | Hargreaves et al., 2009a,b |
| Composted farmyard manure | 1.2 | 0.38 | 0.44 | Chan et al., 2007a | Compost | Kenya | 90 | Nekesa et al., 2007 |
| Composted farmyard manure | 1.9 | 0.65 | 0.67 | Hargreaves et al., 2009a,b |  |  |  |  |
| **Vermicompost** | | | | |  |  |  |  |
| Straw, manure, food waste | 2.6 | 0.9 | 2.4 | Suthar, 2007 | Vermicompost | Kenya | 350 | Ndung’u, 2016 |
| Paper waste | 1.0 | 2.7 | 6.2 | Arancon et al., 2004 | Vermicompost | Kenya | 500 | Strathmore University, 2017 |
| Food waste | 1.3 | 2.7 | 9.2 | Arancon et al., 2004 | Vermicompost | Vietnam | 100 | Viet D.E.L.T.A Industrial Co., LTD; Alibaba |
| Worm castings | 1.5 | 1.1 | 1.08 | Penhallegon, R., 2003 | Vermicompost | India | 31.11 | Vermico company |
| Worm castings | 3.2 | 0.5 | 1.25 | Traunfeld and Nibali, 2013 | Vermicompost | China | 225 | Shijiazhuang Hanhao Trade Co., Ltd; Alibaba |
| **Biochar** | | | | |  |  |  |  |
| Poultry litter | 5.2 | 0.58 | 2.5 | Singh et al., 2010 | Biochar (70% derived from plant biomass) | Global mean | 2650 | Jirka and Tomlinson, 2014 |
| Cow manure | 1.4 | 0.44 | 2.6 | Singh et al., 2010 | Biochar | Philippines | 90 | Jirka and Tomlinson, 2014 |
| E. Saligna wood | 1.4 | 0.01 | 0.18 | Singh et al., 2010 | Biochar | UK | 8850 | Jirka and Tomlinson, 2014 |
| HSW 500 °C | 3.0 | 2.5 | 2.6 | Krounbi et al. (2018) | Biochar (woody feedstock) | USA | 525 | Wakefield Biochar |
| **Miscellaneous organic amendments** | | | | |  |  |  |  |
| Biosolids (Milorganite) | 6.0 | 1.75 | 0 | Wallmart | Biosolids | USA | 257.94 | Wallmart |
| Alfalfa meal | 2.5 | 0.22 | 1.66 | Penhallegon, 2003 | Alfalfa meal | USA | 264.75 | USDA market news, 6/20/2017 |
| Alfalfa meal | 3.0 | 0.44 | 1.66 | Traunfeld and Nibali, 2013 |  |  |  |  |
| Cottonseed meal | 5.0 | 0.87 | 0.44 | Penhallegon, 2003 | Cottonseed meal | USA | 212.5 | USDA market news, 6/20/2017 |
| Cottonseed meal | 6.0 | 0.87 | 0.44 | Traunfeld and Nibali, 2013 |  |  |  |  |
| Soy meal | 6.5 | 0.65 | 1.99 | Penhallegon, 2003 | Soy meal | USA | 307.86 | USDA market news, 6/20/2017 |
| Soy meal | 7.0 | 0.87 | 0.83 | Traunfeld and Nibali, 2013 |  |  |  |  |
| Bone meal | 1.0 | 4.8 | 0 | Traunfeld and Nibali, 2013 | Bone meal | USA | 247.1 | USDA market news, 6/20/2017 |
| Feather meal | 15.0 | 0 | 0 | Penhallegon, 2003 |  |  |  |  |

Supplementary Table 6

R software packages used for data organization, analysis, and presentation.

| Count | R package | Authors |
| --- | --- | --- |
| 1 | broom | Robinson (2017) |
| 2 | cowplot | Wilke (2017) |
| 3 | data.table | Dowle and Srinivasan (2017) |
| 4 | devtools | Wickham and Chang (2017) |
| 5 | dplyr | Wickham et al. (2017) |
| 6 | ggplot2 | Wickham (2009) |
| 7 | ggpmisc | Aphalo. (2016) |
| 8 | grid | R Core Team (2017) |
| 9 | lattice | Sarkar (2008) |
| 10 | lsmeans | Lenth (2016) |
| 11 | multcompView | Graves et al. (2015) |
| 12 | plotly | Sievert et al. (2017) |
| 13 | plyr | Wickham (2011) |
| 14 | quantreg | Koenker (2018) |
| 15 | reshape2 | Wickham (2007) |
| 16 | stringr | Wickham (2017) |
| 17 | tidyverse | Wickham (2017) |
| 18 | xlsx | Dragulescu (2014) |
| 19 | xlsxjars | Dragulescu (2014) |

Supplementary Table 7

Fecal pathogens in HSW sanitized through various methods at different highest heating temperatures (HHT).

| Treatment | HHT (°C) | Intestinal ova and parasites | *Salmonella* species | *Shigella* species | *Aeromonas* species | *E. coli* serotype 157 | *Yersinia enterocolitica* |
| --- | --- | --- | --- | --- | --- | --- | --- |
| Raw | 25 | Entamoeba histolytica, Giardia lamblia cysts | ND^a^ | ND | ND | ND | ND |
| Sun-dried | 40 | ND | ND | ND | ND | ND | ND |
| Autoclaved | 105 | ND | ND | ND | ND | ND | ND |
| Composted | 60 | ND | ND | ND | ND | ND | ND |
| Torrefied | 200 | ND | ND | ND | ND | ND | ND |
| Pyrolyzed | 300 | ND | ND | ND | ND | ND | ND |
| Pyrolyzed | 400 | ND | ND | ND | ND | ND | ND |
| Pyrolyzed | 500 | ND | ND | ND | ND | ND | ND |
| Pyrolyzed | 600 | ND | ND | ND | ND | ND | ND |

^a^ND = not detected

Supplementary Table 8

Total polyaromatic hydrocarbons (PAHs) in HSW amendments.

|  | Highest heating temperature (°C) | | | |
| --- | --- | --- | --- | --- |
|  | 60 (compost) | 300 | 500 | 700 |
| Congener | (μg/kg) | | | |
| Acenaphthene | ND^a^ | ND | 8.2 | 4.56 |
| Acenaphthylene | ND | 373 | 2.56 | ND |
| Anthracene | ND | ND | 26.2 | ND |
| Benzo(a)anthracene | 6.7 | ND | 9.63 | ND |
| Benzo(a)pyrene | 6.7 | ND | 3.22 | ND |
| Benzo(b)fluoranthene | 6 | ND | 3.51 | ND |
| Benzo(g,h,i)perylene | ND | ND | 2.26 | ND |
| Benzo(k)fluoranthene | 6 | ND | 2.88 | ND |
| Chrysene | 6.7 | ND | 19.9 | ND |
| Dibenzo(a,h)anthracene | ND | ND | ND | ND |
| Fluoranthene | 7.3 | ND | 18.1 | ND |
| Fluorene | ND | 186 | 20.4 | 4.26 |
| Indeno(1,2,3-cd)pyrene | ND | ND | 1.84 | ND |
| 2-Methylnaphthalene | ND | 261 | 205 | 14.9 |
| Naphthalene | ND | 122 | 1160 | 21.6 |
| Phenanthrene | 8 | ND | 129 | 8.85 |
| Pyrene | 8.7 | ND | 19.8 | ND |
| **Total PAHs** | 56.1 | 942 | 1632.5 | 54.17 |

^a^ ND = not detected

Supplementary Table 9

Concentrations of polychlorinated bi-phenyls (PCBs) in HSW amendments.

|  | Highest heating temperature (°C) | | | | | |
| --- | --- | --- | --- | --- | --- | --- |
|  | 60 (compost) | | 300 | 500 | 700 | |
| Congener type | | (μg/kg) | | | |  |
| Monochlorobiphenyls | 0.119 | | 0.107 | 0.174 | 0.256 | |
| Dichlorobiphenyls | 0.186 | | 0.522 | 0.656 | 1.170 | |
| Trichlorobiphenyls | 0.205 | | 0.205 | 0.387 | 0.626 | |
| Tetrachlorobiphenyls | 0.138 | | ND^a^ | ND | 0.292 | |
| Pentachlorobiphenyls | 0.748 | | 0.077 | ND | 0.243 | |
| Hexachlorobiphenyls | 0.564 | | ND | ND | ND | |
| Heptachlorobiphenyls | ND | | ND | ND | ND | |
| Octachlorobiphenyls | ND | | ND | ND | ND | |
| Nonachlorobiphenyls | ND | | ND | ND | ND | |
| Decachlorobiphenyls | ND | | ND | ND | ND | |
| **Total PCBs** | **1.96** | | **0.91** | **1.22** | **2.59** | |

^a^ ND = not detected

Supplementary Table 10

Concentrations of PCCD/Fs found in HSW amendments and associated TEFs for each congener.

|  |  | | Highest heating temperature (°C) | | | |  |
| --- | --- | --- | --- | --- | --- | --- | --- |
|  | TEF | | 60 (compost) | 300 | 500 | 700 |  |
| Congener type | | (μg/kg) | | | | | |
| 2,3,7,8 TCDF | 0.1 | | 2 | ND^a^ | ND | ND |  |
| Total TCDF |  | | 63 | ND | ND | ND |  |
| 2,3,7,8 TCDD | 1 | |  | ND | ND | ND |  |
| Total TCDD |  | | 26 | ND | ND | ND |  |
| 1,2,3,7,8 PeCDF | 0.03 | |  | ND | ND | ND |  |
| 2,3,4,7,8 PeCDF | 0.3 | |  | ND | ND | ND |  |
| Total PeCDF |  | | 17 | ND | ND | ND |  |
| 1,2,3,7,8 PeCDD | 1 | |  | ND | ND | ND |  |
| Total PeCDD |  | | 8 | ND | ND | ND |  |
| 1,2,3,4,7,8 HxCDF | 0.1 | |  | ND | ND | ND |  |
| 1,2,3,6,7,8 HxCDF | 0.1 | |  | ND | ND | ND |  |
| 2,3,4,6,7,8 HxCDF | 0.1 | |  | ND | ND | ND |  |
| 1,2,3,7,8,9 HxCDF | 0.1 | |  | ND | ND | ND |  |
| Total HxCDF |  | | 6.4 | ND | ND | ND |  |
| 1,2,3,4,7,8 HxCDD | 0.1 | |  | ND | ND | ND |  |
| 1,2,3,6,7,8 HxCDD | 0.1 | |  | ND | ND | ND |  |
| 1,2,3,7,8,9 HxCDD | 0.1 | |  | ND | ND | ND |  |
| Total HxCDD |  | | 17 | ND | ND | ND |  |
| 1,2,3,4,6,7,8 HpCDF | 0.01 | | 10 | ND | ND | ND |  |
| 1,2,3,4,7,8,9 HpCDF | 0.01 | |  | ND | ND | ND |  |
| Total HpCDF |  | | 19 | ND | ND | ND |  |
| 1,2,3,4,6,7,8 HpCDD | 0.01 | | 42 | ND | ND | ND |  |
| Total HpCDD |  | | 80 | ND | ND | ND |  |
| OCDF | 0.0003 | | 27 | ND | ND | ND |  |
| OCDD | 0.0003 | | 820 | ND | ND | ND |  |
| **Total TEQ** |  | | **0.97** | **0** | **0** | **0** |  |

^a^ ND = not detected

Supplementary Table 11

Median quantile value (p = 0.5) of agronomically-beneficial components in HSW amendments per megagram (Mg) of dry, unsanitized HSW (feedstock) and sanitized HSW (amendment). Agronomic components include plant-available N (NH_4_^+^+ NO_3_^-^), P, K, Ca, Mg, S, micronutrients (B, Cu, Mn, Zn) the contribution of the CEC toward retention of K^+^, Ca^2+^, and Mg^2+^, CaCO_3_ equivalency, and BC_+100_.

|  | HSW highest heating temperature (°C) | | | | | | |
| --- | --- | --- | --- | --- | --- | --- | --- |
| Agronomic component^a^ | 60 (compost) | 200 | 300 | 400 | 500 | 600 | 700 |
|  | (USD/Mg feedstock) | | | | | | |
| NH_4_^+^+ NO_3_^-^-N | 0.5 ± 0.0 | 1.8 ± 0.0 | 0.0 ± 0.0 | 0.0 ± 0.0 | 0.0 ± 0.0 | 0.0 ± 0.0 | 0.0 ± 0.0 |
| P | 5.2 ± 0.3 | 50.2 ± 3.4 | 27.5 ± 1.3 | 23.4 ± 1.5 | 23.0 ± 1.2 | 22.6 ± 1.4 | 18.3 ± 0.7 |
| K | 8.4 ± 0.4 | 73.4 ± 2.3 | 49.4 ± 1.9 | 44.7 ± 1.9 | 44.6 ± 1.4 | 42.5 ± 1.2 | 33.5 ± 1.0 |
| Ca | 0.4 ± 0.0 | 0.6 ± 0.0 | 0.3 ± 0.0 | 0.3 ± 0.0 | 0.2 ± 0.0 | 0.2 ± 0.0 | 0.2 ± 0.0 |
| Mg | 0.3 ± 0.0 | 1.6 ± 0.1 | 0.9 ± 0.0 | 0.8 ± 0.1 | 0.9 ± 0.1 | 0.9 ± 0.1 | 0.6 ± 0.0 |
| S | 1.2 ± 0.1 | 6.3 ± 0.4 | 1.4 ± 0.1 | 1.3 ± 0.1 | 1.4 ± 0.1 | 1.4 ± 0.1 | 1.4 ± 0.0 |
| B | 0.0 ± 0.0 | 0.0 ± 0.0 | 0.0 ± 0.0 | 0.0 ± 0.0 | 0.0 ± 0.0 | 0.0 ± 0.0 | 0.0 ± 0.0 |
| Cu | 0.0 ± 0.0 | 0.1 ± 0.0 | 0.0 ± 0.0 | 0.0 ± 0.0 | 0.0 ± 0.0 | 0.1 ± 0.0 | 0.2 ± 0.0 |
| Mn | 0.2 ± 0.0 | 0.2 ± 0.0 | 0.1 ± 0.0 | 0.1 ± 0.0 | 0.1 ± 0.0 | 0.1 ± 0.0 | 0.0 ± 0.0 |
| Zn | 0.1 ± 0.0 | 0.2 ± 0.0 | 0.0 ± 0.0 | 0.0 ± 0.0 | 0.0 ± 0.0 | 0.0 ± 0.0 | 0.1 ± 0.0 |
| Micronutrients (B+Cu+Zn+Mn) | 0.3 ± 0.0 | 0.4 ± 0.0 | 0.1 ± 0.0 | 0.1 ± 0.0 | 0.1 ± 0.0 | 0.2 ± 0.0 | 0.3 ± 0.0 |
| K^+^ (CEC) | 8.4 ± 0.3 | 15.0 ± 1.0 | 20.4 ± 1.3 | 13.8 ± 0.5 | 9.4 ± 0.7 | 5.0 ± 0.4 | 5.2 ± 0.3 |
| Ca^2+^ (CEC) | 0.3 ± 0.0 | 0.1 ± 0.0 | 0.1 ± 0.0 | 0.0 ± 0.0 | 0.0 ± 0.0 | 0.0 ± 0.0 | 0.0 ± 0.0 |
| Mg^2+^ (CEC) | 0.1 ± 0.0 | 0.1 ± 0.0 | 0.1 ± 0.0 | 0.1 ± 0.0 | 0.1 ± 0.0 | 0.0 ± 0.0 | 0.0 ± 0.0 |
| CEC (K^+^+Ca^2+^+Mg^2+^) | 8.8 ± 0.3 | 15.1 ± 1.0 | 20.6 ± 1.3 | 13.9 ± 0.5 | 9.5 ± 0.7 | 5.0 ± 0.4 | 5.3 ± 0.3 |
| CaCO_3_ | 2.4 ± 0.8 | 0.5 ± 0.8 | 1.4 ± 0.1 | 2.6 ± 0.3 | 3.4 ± 0.1 | 2.3 ± 0.0 | 3.9 ± 0.2 |
| BC_+100_ | 0.1 ± 0.1 | 0.4 ± 0.2 | 1.1 ± 0.1 | 1.2 ± 0.0 | 1.3 ± 0.1 | 1.3 ± 0.0 | 1.3 ± 0.0 |
| Sum | 26.4 ± 2.4 | 144.2 ± 5.9 | 101.4 ± 3.7 | 87.0 ± 3.2 | 83.1 ± 2.6 | 74.8 ± 2.4 | 63.4 ± 2.0 |
|  | (USD/Mg amendment) | | | | | | |
| NH_4_^+^+ NO_3_^-^-N | 1.1 ± 0.0 | 2.0 ± 0.0 | 0.1 ± 0.0 | 0.0 ± 0.0 | 0.0 ± 0.0 | 0.0 ± 0.0 | 0.0 ± 0.0 |
| P | 10.4 ± 0.7 | 55.7 ± 3.8 | 48.2± 2.3 | 51.9 ± 3.3 | 59 ± 3.2 | 66.4 ± 4.2 | 55.3 ± 2.2 |
| K | 16.8 ± 0.9 | 81.5 ± 2.6 | 86.7 ± 3.4 | 99.3 ± 4.2 | 114.3 ± 3.5 | 124.9 ± 3.4 | 101.4 ± 3.1 |
| Ca | 0.9 ± 0.1 | 0.6 ± 0.0 | 0.5 ± 0.0 | 0.6 ± 0.1 | 0.6 ± 0.0 | 0.5 ± 0.0 | 0.7 ± 0.0 |
| Mg | 0.6 ± 0.0 | 1.7 ± 0.1 | 1.6 ± 0.1 | 1.8 ± 0.1 | 2.3 ± 0.2 | 2.6 ± 0.2 | 1.7 ± 0.1 |
| S | 0.1 ± 0.0 | 0.2 ± 0.0 | 0.1 ± 0.0 | 0.1 ± 0.0 | 0.1 ± 0.0 | 0.1 ± 0.0 | 0.1 ± 0.0 |
| Cu | 0.0 ± 0.0 | 0.1 ± 0.0 | 0.0 ± 0.0 | 0.0 ± 0.0 | 0.1 ± 0.0 | 0.2 ± 0.0 | 0.6 ± 0.1 |
| B | 0.0 ± 0.0 | 0.0 ± 0.0 | 0.0 ± 0.0 | 0.0 ± 0.0 | 0.0 ± 0.0 | 0.0 ± 0.0 | 0.0 ± 0.0 |
| Mn | 0.4 ± 0.0 | 0.2 ± 0.0 | 0.1 ± 0.0 | 0.2 ± 0.0 | 0.2 ± 0.0 | 0.2 ± 0.0 | 0.1 ± 0.0 |
| Zn | 0.1 ± 0.0 | 0.3 ± 0.0 | 0.0 ± 0.0 | 0.1 ± 0.0 | 0.1 ± 0.0 | 0.1 ± 0.0 | 0.1 ± 0.0 |
| Micronutrients (B+Cu+Mn+Zn) | 0.5 ± 0.0 | 0.5 ± 0.0 | 0.2 ± 0.0 | 0.3 ± 0.0 | 0.4 ± 0.0 | 0.5 ± 0.0 | 0.9 ± 0.1 |
| K^+^ (CEC) | 16.8 ± 0.6 | 16.6 ± 1.1 | 35.9 ± 2.2 | 30.7 ± 1.1 | 24.1 ± 1.7 | 14.6 ± 1.3 | 15.8 ± 1.0 |
| Ca^2+^ (CEC) | 0.6 ± 0.0 | 0.1 ± 0.0 | 0.1 ± 0.0 | 0.1 ± 0.0 | 0.1 ± 0.0 | 0.0 ± 0.0 | 0.1 ± 0.0 |
| Mg^2+^ (CEC) | 0.2 ± 0.0 | 0.1 ± 0.0 | 0.2 ± 0.0 | 0.2 ± 0.0 | 0.2 ± 0.0 | 0.1 ± 0.0 | 0.1 ± 0.0 |
| CEC (K^+^+Ca^2+^+Mg^2+^) | 17.6 ± 0.6 | 16.8 ± 1.1 | 36.2 ± 2.2 | 31.0 ± 1.1 | 24.3 ± 1.7 | 14.7 ± 1.3 | 15.9 ± 1.0 |
| CaCO_3_ | 4.7 ± 1.5 | 0.6 ± 0.8 | 2.4 ± 0.2 | 5.8 ± 0.8 | 8.6 ± 0.2 | 6.6 ± 0.0 | 11.9 ± 0.7 |
| BC_+100_ | 0.1 ± 0.2 | 0.5 ± 0.2 | 1.92 ± 0.09 | 2.6 ± 0.0 | 3.4 ± 0.2 | 3.7 ± 0.0 | 4.1 ± 0.0 |
| Sum | 52.7 ± 4.9 | 160.2 ± 6.5 | 177.8 ± 6.5 | 193.4 ± 7.2 | 212.9 ± 6.7 | 220.0 ± 7.2 | 192.0 ± 6.0 |

^a^ Prices for N (NH_4_^+^+ NO_3_^-^), P, K, Ca, and CaCO_3_ include products from both African and international markets. International prices were used for Mg, S, and micronutrients, while prices for BC_+100_ refer to CO_2_ discount rates across 30 countries. Median (0.5 quantile) price values have been used for calculations ± standard deviation.

Supplementary Table 12

Coefficients of regression, slope (β) and intercept (α), of quantiles of the monetary value of agronomic components in HSW versus highest heating temperature (HHT).

| Element |  | Quantile | Value ± std error | t value | p value |
| --- | --- | --- | --- | --- | --- |
| NH_4_^+^+ NO_3_^-^ | α | 0.1 | 849.5 ± 32.5 | 26.16 | 0.00 |
|  | β |  | -1.6 ± 0.1 | -23.09 | 0.00 |
|  | α | 0.25 | 1719.6 ± 98.5 | 17.46 | 0.00 |
|  | β |  | -3.2 ± 0.2 | -15.32 | 0.00 |
|  | α | 0.5 | 6364.8 ± 380.8 | 16.71 | 0.00 |
|  | β |  | -10.6 ± 0.6 | -16.66 | 0.00 |
|  | α | 0.75 | 18153.4 ± 450.3 | 40.31 | 0.00 |
|  | β |  | -25.9 ± 0.6 | -40.31 | 0.00 |
|  | α | 0.9 | 32871.2 ± 1409.2 | 23.33 | 0.00 |
|  | β |  | -46.9 ± 2.0 | -23.31 | 0.00 |
| P | α | 0.1 | 31602.2 ± 1730.4 | 18.26 | 0.00 |
|  | β |  | 339.5 ± 9.2 | 36.94 | 0.00 |
|  | α | 0.25 | 74950.3 ± 5720.1 | 13.1 | 0.00 |
|  | β |  | 464.8 ± 21.09 | 22.04 | 0.00 |
|  | α | 0.5 | 142790.4 ± 19655.0 | 7.26 | 0.00 |
|  | β |  | 799.3 ± 48.3 | 16.54 | 0.00 |
|  | α | 0.75 | 394929.9 ± 21629.7 | 18.26 | 0.00 |
|  | β |  | 834.4 ± 87.0 | 9.59 | 0.00 |
|  | α | 0.9 | 425794.2 ± 152072.4 | 2.8 | 0.00 |
|  | β |  | 2829.1 ± 269.7 | 10.49 | 0.00 |
| K | α | 0.1 | 27184.3 ± 14632.8 | 1.86 | 0.06 |
|  | β |  | 457.3 ± 58.7 | 7.79 | 0.00 |
|  | α | 0.25 | 72871.1 ± 9430.3 | 7.73 | 0.00 |
|  | β |  | 1021.8 ± 81.5 | 12.54 | 0.00 |
|  | α | 0.5 | 274118.23 ± 64141.0 | 4.27 | 0.00 |
|  | β |  | 1505.9 ± 159.3 | 9.45 | 0.00 |
|  | α | 0.75 | 333486.3 ± 59122.8 | 5.64 | 0.00 |
|  | β |  | 2796.4 ± 257.7 | 10.85 | 0.00 |
|  | α | 0.9 | 1476473.7 ± 192463.7 | 7.67 | 0.00 |
|  | β |  | 3318.7 ± 436.8 | 7.6 | 0.00 |
| Ca | α | 0.1 | 0.3 ± 0.1 | 3.05 | 0.00 |
|  | β |  | 0.0 ± 0.0 | 0 | 1.00 |
|  | α | 0.25 | 0.6 ± 0.1 | 7.21 | 0.00 |
|  | β |  | 0.0 ± 0.0 | -1.47 | 0.15 |
|  | α | 0.5 | 0.7 ± 0.1 | 5.27 | 0.00 |
|  | β |  | 0.0 ± 0.0 | -0.78 | 0.44 |
|  | α | 0.75 | 1.3 ± 0.3 | 5.27 | 0.00 |
|  | β |  | 0.0 ± 0.0 | -0.88 | 0.38 |
|  | α | 0.9 | 1.6 ± 0.2 | 7.73 | 0.00 |
|  | β |  | 0.0 ± 0.0 | -1.38 | 0.17 |
| Mg | α | 0.1 | 0.3 ± 0.1 | 3.44 | 0.00 |
|  | β |  | 0.0 ± 0.0 | 5.55 | 0.00 |
|  | α | 0.25 | 0.5 ± 0.1 | 4.03 | 0.00 |
|  | β |  | 0.0 ± 0.0 | 7.13 | 0.00 |
|  | α | 0.5 | 0.6 ± 0.3 | 1.91 | 0.06 |
|  | β |  | 0.0 ± 0.0 | 3.51 | 0.00 |
|  | α | 0.75 | 1.0 ± 0.3 | 3.43 | 0.00 |
|  | β |  | 0.0 ± 0.0 | 4.35 | 0.00 |
|  | α | 0.9 | 1.5 ± 0.4 | 3.45 | 0.00 |
|  | β |  | 0.0 ± 0.0 | 2.18 | 0.03 |
| S | α | 0.1 | 0.1 ± 0.0 | 1.2E+16 | 0.00 |
|  | β |  | 0.0 ± 0.0 | -0.72 | 0.48 |
|  | α | 0.25 | 0.1 ± 0.0 | 5.82E+15 | 0.00 |
|  | β |  | 0.0 ± 0.0 | -0.31 | 0.75 |
|  | α | 0.5 | 0.1 ± 0.0 | 1.14E+16 | 0.00 |
|  | β |  | 0.0 ± 0.0 | 0 | 1.00 |
|  | α | 0.75 | 0.1 ± 0.1 | 1.43 | 0.16 |
|  | β |  | 0.0 ± 0.0 | 0 | 1.00 |
|  | α | 0.9 | 0.2 ± 0.0 | 1.23E+16 | 0.00 |
|  | β |  | 0.0 ± 0.0 | -1.1E+15 | 0.00 |
| Micronutrients (B+Cu+Mn+Zn) | α | 0.1 | 1.6 ± 0.5 | 2.99 | 0.00 |
|  | β |  | 0.0 ± 0.0 | 0.56 | 0.57 |
|  | α | 0.25 | 1.9 ± 0.7 | 2.74 | 0.01 |
|  | β |  | 0.0 ± 0.0 | 0.87 | 0.39 |
|  | α | 0.5 | 4.0 ± 0.6 | 6.98 | 0.00 |
|  | β |  | 0.0 ± 0.0 | -1.43 | 0.16 |
|  | α | 0.75 | 5.0 ± 0.4 | 14.48 | 0.00 |
|  | β |  | 0.0 ± 0.0 | -3.21 | 0.00 |
|  | α | 0.9 | 5.8 ± 0.3 | 17.99 | 0.00 |
|  | β |  | 0.0 ± 0.0 | -4.36 | 0.00 |
| K^+^ (CEC) | α | 0.1 | 50219.1 ± 4211.5 | 11.92 | 0.00 |
|  | β |  | -10.8 ± 8.8 | -1.24 | 0.22 |
|  | α | 0.25 | 128810.2 ± 6938.7 | 18.56 | 0.00 |
|  | β |  | -27.4 ± 16.4 | -1.67 | 0.10 |
|  | α | 0.5 | 225458.0 ± 12759.2 | 17.67 | 0.00 |
|  | β |  | -50.7 ± 23.5 | -2.16 | 0.03 |
|  | α | 0.75 | 453749.0 ± 34420.3 | 13.18 | 0.00 |
|  | β |  | -158.5 ± 91.2 | -1.74 | 0.08 |
|  | α | 0.9 | 616957.9 ± 88275.1 | 6.99 | 0.00 |
|  | β |  | -110.5 ± 142.8 | -0.77 | 0.44 |
| Ca^2+^ (CEC) | α | 0.1 | 0.2 ± 0.1 | 2.02 | 0.05 |
|  | β |  | 0.0 ± 0.0 | -1.97 | 0.05 |
|  | α | 0.25 | 0.2 ± 0.0 | 7.0 | 0.00 |
|  | β |  | 0.0 ± 0.0 | -3.77 | 0.00 |
|  | α | 0.5 | 0.3 ± 0.1 | 2.76 | 0.01 |
|  | β |  | 0.0 ± 0.0 | -1.88 | 0.06 |
|  | α | 0.75 | 0.4 ± 0.1 | 3.66 | 0.00 |
|  | β |  | 0.0 ± 0.0 | -2.63 | 0.01 |
|  | α | 0.9 | 1.0 ± 0.3 | 3.26 | 0.00 |
|  | β |  | 0.0 ± 0.0 | -2.93 | 0.00 |
| Mg^2+^ (CEC) | α | 0.1 | 0.1 ± 0.0 | 8.5 | 0.00 |
|  | β |  | 0.0 ± 0.0 | 0 | 1.00 |
|  | α | 0.25 | 0.1 ± 0.0 | 3.62 | 0.00 |
|  | β |  | 0.0 ± 0.0 | 0 | 1.00 |
|  | α | 0.5 | 0.2 ± 0.0 | 6.41 | 0.00 |
|  | β |  | 0.0 ± 0.0 | -3.32 | 0.00 |
|  | α | 0.75 | 0.3 ± 0.0 | 8.22 | 0.00 |
|  | β |  | 0.1 ± 0.1 | -3.25 | 0.00 |
|  | α | 0.9 | 0.3 ± 0.1 | 4.96 | 0.00 |
|  | β |  | 0.0 ± 0.0 | -1.82 | 0.07 |
| CaCO_3_ | α | 0.1 | -1.3 ± 0.4 | -3.20 | 0.00 |
|  | β |  | 0.0 ± 0.1 | 5.81 | 0.00 |
|  | α | 0.25 | -1.7 ± 0.7 | -2.53 | 0.01 |
|  | β |  | 0.0 ± 0 | 5.34 | 0.00 |
|  | α | 0.5 | -0.2 ± 1.8 | -0.11 | 0.91 |
|  | β |  | 0.0 ± 0 | 3.97 | 0.00 |
|  | α | 0.75 | 3.4 ± 1.7 | 2.02 | 0.05 |
|  | β |  | 0.0 ± 0 | 3.91 | 0.00 |
|  | α | 0.9 | 7.8 ± 2.1 | 3.68 | 0.00 |
|  | β |  | 0.0 ± 0 | 4.76 | 0.00 |
| BC_+100_ | α | 0.1 | -0.1 ± 0.1 | -1.24 | 0.22 |
|  | β |  | 0.0 ± 0.0 | 3.67 | 0.00 |
|  | α | 0.25 | -0.2 ± 0.1 | -2.53 | 0.01 |
|  | β |  | 0.0 ± 0.0 | 6.06 | 0.00 |
|  | α | 0.5 | -0.3 ± 0.1 | -2.52 | 0.01 |
|  | β |  | 0.0 ± 0.0 | 5.1 | 0.00 |
|  | α | 0.75 | -0.6 ± 0.2 | -2.54 | 0.01 |
|  | β |  | 0.1 ± 0.0 | 4.11 | 0.00 |
|  | α | 0.9 | -1.1 ± 0.3 | -3.96 | 0.00 |
|  | β |  | 0.0 ± 0.0 | 7.87 | 0.00 |

Supplementary Table 13

Value of total N, P, and K in HSW sanitized at different HHTs benchmarked against the median market price of nine commercial organic amendments according to respective concentrations of total N, P, and K (Supplementary Table 4, Supplementary Table 5). The average value of HSW amendments, shown at the bottom of the table, does not include the value benchmarked against biochar.

|  |  | Highest heating temperature (°C) | | | | | | |
| --- | --- | --- | --- | --- | --- | --- | --- | --- |
| Nutrient | 60 (compost) | 200 | 300 | 400 | 500 | 600 | 700 |  |
|  |  | (USD/Mg amendment) | | | | | | |
| Manure | N | 3.1 | 6.5 | 8.6 | 8.5 | 7.2 | 6.7 | 6 |
|  | P | 4.6 | 6.7 | 11.9 | 15.1 | 14.7 | 17.6 | 18.4 |
|  | K | 2.3 | 4.9 | 5 | 4.6 | 12.4 | 5.6 | 16.6 |
|  | Sum N+P+K | 10 | 18.1 | 25.6 | 28.2 | 34.3 | 29.9 | 41 |
| Compost | N | 18.7 | 38.7 | 51.5 | 50.6 | 42.9 | 40 | 35.7 |
|  | P | 27.6 | 39.7 | 71.2 | 90.4 | 87.6 | 105.1 | 110 |
|  | K | 13.6 | 29.4 | 29.9 | 27.7 | 74 | 33.7 | 99.1 |
|  | Sum N+P+K | 59.9 | 107.9 | 152.7 | 168.6 | 204.6 | 178.7 | 244.7 |
| Vermicompost | N | 21.8 | 45.2 | 60.1 | 59 | 50 | 46.7 | 41.6 |
|  | P | 32.2 | 46.4 | 83.1 | 105.5 | 102.2 | 122.6 | 128.3 |
|  | K | 15.9 | 34.3 | 34.9 | 32.3 | 86.4 | 39.3 | 115.6 |
|  | Sum N+P+K | 69.8 | 125.8 | 178.1 | 196.7 | 238.6 | 208.5 | 285.5 |
| Milorganite | N | 35.3 | 73.3 | 97.4 | 95.6 | 81.1 | 75.7 | 67.5 |
|  | P | 52.1 | 75.2 | 134.7 | 171 | 165.7 | 198.7 | 207.9 |
|  | K | 25.8 | 55.6 | 56.6 | 52.3 | 140 | 63.6 | 187.4 |
|  | Sum N+P+K | 113.2 | 204 | 288.7 | 318.9 | 386.8 | 338 | 462.8 |
| Soybean meal | N | 38.3 | 79.4 | 105.6 | 103.6 | 87.9 | 82 | 73.1 |
|  | P | 56.5 | 81.5 | 145.9 | 185.3 | 179.6 | 215.3 | 225.4 |
|  | K | 27.9 | 60.2 | 61.4 | 56.7 | 151.7 | 69 | 203.1 |
|  | Sum N+P+K | 122.7 | 221.1 | 312.9 | 345.6 | 419.2 | 366.3 | 501.6 |
| Cottonseed meal | N | 35.7 | 73.9 | 98.3 | 96.5 | 81.9 | 76.4 | 68.1 |
|  | P | 52.6 | 75.8 | 135.9 | 172.5 | 167.2 | 200.4 | 209.8 |
|  | K | 26 | 56.1 | 57.1 | 52.8 | 141.2 | 64.2 | 189.1 |
|  | Sum N+P+K | 114.2 | 205.8 | 291.3 | 321.7 | 390.3 | 341 | 467 |
| Alfalfa meal | N | 53.5 | 111 | 147.6 | 144.9 | 122.9 | 114.7 | 102.2 |
|  | P | 79 | 113.9 | 204 | 259.1 | 251.1 | 301 | 315.1 |
|  | K | 39.1 | 84.2 | 85.8 | 79.2 | 212.1 | 96.4 | 284 |
|  | Sum N+P+K | 171.6 | 309.1 | 437.4 | 483.2 | 586.2 | 512.1 | 701.3 |
| Bone meal | N | 16.6 | 34.3 | 45.6 | 44.8 | 38 | 35.4 | 31.6 |
|  | P | 24.4 | 35.2 | 63.1 | 80.1 | 77.6 | 93.1 | 97.4 |
|  | K | 12.1 | 26 | 26.5 | 24.5 | 65.6 | 29.8 | 87.8 |
|  | Sum N+P+K | 53 | 95.5 | 135.2 | 149.3 | 181.2 | 158.3 | 216.8 |
| *Biochar (Poultry and cattle manure, wood, HSW 500 °C) | N | 245.8 | 509.6 | 677.6 | 665 | 564.3 | 526.3 | 469.2 |
|  | P | 362.5 | 522.8 | 936.5 | 1189.2 | 1152.6 | 1381.8 | 1446.3 |
|  | K | 179.3 | 386.4 | 393.8 | 363.6 | 973.6 | 442.6 | 1303.5 |
|  | Sum N+P+K | 787.5 | 1418.8 | 2007.9 | 2217.8 | 2690.5 | 2350.7 | 3219.1 |
| **AVERAGE HSW value (excluding biochar)** | | **79.4** | **143.0** | **202.4** | **223.6** | **271.2** | **237.0** | **324.5** |

**References**

1. Aphalo, O.J., 2016. Learn R ...as you learnt your mother tongue. Leanpub, Helsinki.
2. Arancon, N.Q., Edwards, C.A., Bierman, P., Welch, C., Metzger, J.D., 2004. Influences of vermicomposts on field strawberries: 1. Effects on growth and yields. Bioresour. Technol. 93 (2), 145-153.
3. Carpenter-Boggs, L., Reganold, J.P., Kennedy, A.C., 2000. Effects of biodynamic preparations on compost development. Biol. Agric. Hortic. 17 (4), 313-328.
4. Chan, K.Y., Dorahy, C., Tyler, S., 2007. Determining the agronomic value of composts produced from garden organics from metropolitan areas of New South Wales, Australia. Aust. J. Exp. Agric. 47 (11), 1377-1382.
5. Dowle, M., Srinivasan, A., 2017. data.table: Extension of `data.frame`. R package version 1.10.4-3. https://CRAN.R-project.org/package=data.table (accessed January 2016).
6. Dragulescu, A.A., 2014. xlsx: Read, write, format Excel 2007 and Excel 97/2000/XP/2003 files. R package version 0.5.7. https://CRAN.R-project.org/package=xlsx (accessed January 2016).
7. Dragulescu, A.A., 2014. xlsxjars: Package required POI jars for the xlsx package. R package version 0.6.1. https://CRAN.R-project.org/package=xlsxjars (accessed January 2016).
8. Graves, S., Piepho, H.P., Selzer, L., with help from Dorai-Raj, S., 2015.multcompView: Visualizations of Paired Comparisons. R package version 0.1-7. https://CRAN.R-project.org/package=multcompViews (accessed January 2016).
9. Hargreaves, J.C., Adl, M.S., Warman, P.R., 2009a. Are compost teas an effective nutrient amendment in the cultivation of strawberries? Soil and plant tissue effects. J. Sci. Food Agric. 89 (3), 390-397.
10. Hargreaves, J.C., Adl, M.S., Warman, P.R., 2009b. The effects of municipal solid waste compost and compost tea on mineral element uptake and fruit quality of strawberries. Compost Sci. Util. 17 (2), 85-94.
11. Home Depot website for soil amendments: https://www.homedepot.com/b/Outdoors-Garden-Center-Landscaping-Soils-Soil-Amendments/Compost/N-5yc1vZbx7hZ1z0ludr (accessed June 2017).
12. Ideal Compost Co. website: http://www.idealcompost.com/ (accessed July 2017).
13. Jirka, S., Tomlinson, T., 2014 State of the Biochar Industry. A Survey of Commercial Activity in the Biochar Field, 2014.
14. Kirigia, A., Njoka, J.T., Kinyua, P.I.D., Young, T.P., 2013. Characterizations of livestock manure market and the income contribution of manure trade in Mukogodo, Laikipia, Kenya. African J. Agric. Res. 8 (46), 5864-5871.
15. Koenker, R., 2018. quantreg: Quantile Regression. R package version 5.35. https://CRAN.R-project.org/package=quantreg (accessed January 2016).
16. Krounbi, L., van Es, H., Karanja, N., Lehmann, J., 2018. Nitrogen and phosphorus availability of biologically and thermochemically decomposed human wastes and urine in soils with different texture and pH. Soil Sci. 183 (2), 51-65.
17. Lenth, R.V., 2016. Least-Squares Means: The R Package lsmeans. J. Statistical Software 69 (1), 1-33. doi:10.18637/jss.v069.i01 (accessed January 2016).
18. Montana State University. "Manure 'Smells Like Money' As Energy Costs Rise." ScienceDaily. ScienceDaily, September 10, 2008. http://www.sciencedaily.com/releases/2008/09/080908225153.htm (accessed August 2017).
19. Ndung’u, A., 2016. Passionate about Dudus. Horti News, Karurui Ventures LTD, Nairobi, Kenya. http://www.hortinews.co.ke/2016/02/09/passionate-about-dudus/ (accessed July 2017).
20. Nekesa, A.O., Okalebo, J.R., Kimetto, J.R., 2007. Adoption of leguminous trees/shrubs, compost and farmyard manure (FYM) as alternatives to improving soil fertility in Trans Nzoia District-Kenya, in: Advances in Integrated Soil Fertility Management in sub-Saharan Africa: Challenges and Opportunities Springer, Dordrecht, pp. 955-960.
21. Njenga, M., Romney, D., Karanja, N., Gathuru, K., Kimani, S., Carsan, S., Frost, W., 2010. Recycling nutrients from organic wastes in Kenya’s capital city, in: African Urban Harvest Springer, New York, NY, pp. 193-212.
22. Otinga, A.N., Pypers, P., Okalebo, J.R., Njoroge, R., Emong’ole, M., Six, L., Vanlauwe, B., Merckx, R., 2013. Partial substitution of phosphorus fertiliser by farmyard manure and its localised application increases agronomic efficiency and profitability of maize production. Field Crops Res. 140, 32-43.
23. Penhallegon, R., 2003. Nitrogen-phosphorus-potassium values of organic fertilizers. Oregon State University Extension Service. Available under http://extension. oregonstate. edu/lane/sites/default/files/documents/lc437organicfertilizersvaluesrev. pdf. (accessed July 2017).
24. R Core Team, 2017. R: A language and environment for statistical computing. R Foundation for Statistical Computing, Vienna, Austria. URL https://www.R-project.org/ (accessed January 2016).
25. Robinson, D., 2017. broom: Convert Statistical Analysis Objects into Tidy Data Frames. R package version 0.4.2. https://CRAN.R-project.org/package=broom (accessed January 2016).
26. Sarkar, D., 2008. Lattice: Multivariate Data Visualization with R. Springer, New York. ISBN 978-0-387-75968-5 (accessed January 2016).
27. Shijiazhuang Hanhao Trade Co., Ltd vermicompost merchant on Alibaba: https://www.alibaba.com/product-detail/vermicompost-organic-fertilizer_60039237614.html?spm=a2700.7724838.2017115.88.30d77071xsoLH1https://www.alibaba.com/product-detail/vermicompost-organic-fertilizer_60039237614.html?spm=a2700.7724838.2017115.88.30d77071xsoLH1 (accessed June 2017).
28. Sievert, C., Parmer, C., Hocking, T., Chamberlain, S., Ram, K., Corvellec, M., Despouy, P., 2017. plotly: Create Interactive Web Graphics via 'plotly.js'. R package version 4.7.1. https://CRAN.R-project.org/package=plotly (accessed January 2016).
29. Singh, B., Singh, B.P., Cowie, A.L., 2010. Characterisation and evaluation of biochars for their application as a soil amendment. Soil Res. 48 (7), 516-525.
30. Strathmore University, 2017. http://www.strathmore.edu/news/agribusiness-vermiculture-the-cultivation-of-earthworms-by-dr-freddie-acosta/ (accessed July 2017).
31. Suthar, S., 2007. Vermicomposting potential of Perionyx sansibaricus (Perrier) in different waste materials. Bioresour. Technol. 98 (6), 1231-1237.
32. Traunfeld, J., Nbali, E., 2013. Soil Amendments and Fertilizers Fertilizing Guidelines Included by Plant Group. University of Maryland Extension, Home and Garden Information Center. (accessed July 2017).
33. http://extension.umd.edu/sites/extension.umd.edu/files/_images/programs/hgic/Publications/HG42_Soil_Amendments_and_Fertilizers.pdf (accessed June 2017).
34. USDA Market News June 20, 2017. https://www.ams.usda.gov/mnreports/ms_gr852.txt (accessed August 2017).
35. Vermico vermicompost merchant: http://www.vermico.com/the-single-largest-producer-of-vermicompost-in-the-world/ (accessed July 2017).
36. Viet D.E.L.T.A Industrial Co., LTD Vermicompost merchant on AliBaba: https://vn1103738867xeoi.trustpass.alibaba.com/company_profile.html#top-nav-bar (accessed June 2017).
37. Wakefield biochar merchant: https://www.wakefieldbiochar.com/ (accessed June 2017).
38. Wallmart price for Milorganite: https://www.walmart.com/ip/0636-Organic-Nitrogen-Fertilizer-36-Pound-An-organic-nitrogen-fertilizer-composed-primarily-of-heat-dried-microbes-By-Milorganite/849093634 (accessed June 2017).
39. Wango, V.N., Mburu, J., Onwong‘a, R., Nyikal, R., 2015. Assessing Profitability of Selected Agro-Ecological Intensification Techniques in: Sorghum and Cassava Based Cropping Systems in: Yatta Sub County, Kenya. Int. J. Sci. Technol. Res. 4 (8), 187-191.
40. Wango, V.N., 2016. An analysis of profitability and factors influencing adoption of agro-ecological intensitification (AEI) techniques in Yatta sub-county, Kenya. Doctoral dissertation, University of Nairobi.
41. Wickham, H., 2007. Reshaping Data with the reshape Package. J. of Statistical Software, 21 (12), 1-20. http://www.jstatsoft.org/v21/i12/ (accessed January 2016).
42. Wickham, H., 2009. ggplot2: Elegant Graphics for Data Analysis. Springer-Verlag New York. http://ggplot2.org (accessed January 2016).
43. Wickham, H., 2011. The Split-Apply-Combine Strategy for Data Analysis. J. of Statistical Software, 40 (1), 1-29. http://www.jstatsoft.org/v40/i01/ (accessed January 2016).
44. Wickham, H., 2017. stringr: Simple, Consistent Wrappers for Common String Operations. R package version 1.2.0. https://CRAN.R-project.org/package=stringr (accessed January 2016).
45. Wickham, H., 2017. tidyverse: Easily Install and Load the 'Tidyverse'. R package version 1.2.1. https://CRAN.R-project.org/package=tidyverse (accessed January 2016).
46. Wickham H., Chang W., 2017. devtools: Tools to Make Developing R Packages Easier. R package version 1.13.4. https://CRAN.R-project.org/package=devtools (accessed January 2016).
47. Wickham, H., Francois, R., Henry, L., Müller, K., 2017. dplyr: A Grammar of Data Manipulation. R package version 0.7.4. https://CRAN.R-project.org/package=dplyr
48. Wilke, C.O., 2017. cowplot: Streamlined Plot Theme and Plot Annotations for 'ggplot2'. R package version 0.9.2. https://CRAN.R-project.org/package=cowplot
49. World Bank, 2015. State and trends of carbon pricing 2015. World Bank Publications.
50. Zaller, J.G., Köpke, U., 2004. Effects of traditional and biodynamic farmyard manure amendment on yields, soil chemical, biochemical and biological properties in a long-term field experiment. Biol. Fert. Soils 40 (4), 222-229.
